# Effects of dietary patterns on the all‐cause mortality and cardiovascular disease mortality in patients with hypertension: A cohort study based on the NHANES database

**DOI:** 10.1002/clc.24118

**Published:** 2023-08-16

**Authors:** Fang Li, Yanping Zhang, Lina Pan, Hui Chen

**Affiliations:** ^1^ Department of Cardiology The Second Hospital of Hebei Medical University Shijiazhuang Hebei People's Republic of China; ^2^ Department of Urological Surgery The Second Hospital of Hebei Medical University Shijiazhuang Hebei People's Republic of China; ^3^ Department of Internal Medicine Wuji County People's Hospital Shijiazhuang Hebei People's Republic of China

**Keywords:** dietary quality assessment tool, hypertension, mortality, NHANES

## Abstract

**Background:**

Hypertension (HTN) patients have higher risk of all‐cause and cardiovascular disease (CVD)‐specific mortality. Dietary patterns have been reported related to the risk of mortality, but their roles in HTN patients is unclear.

**Hypothesis:**

To explore the relationships between different dietary patterns and all‐cause/CVD‐specific mortality and provide dietary guidance for HTN patients' prognosis improvement.

**Methods:**

Data of 27 618 HTN patients were extracted from the National Health and Nutrition Examination Survey (NHANES) database in this retrospective cohort study. The associations between Healthy Eating Index (HEI)‐2015, Alternate Healthy Eating Index (AHEI)‐2010, Dietary Approaches to Stop Hypertension (DASH), and Mediterranean (MED) diet and all‐cause and CVD‐specific mortality were explored using univariate and multivariate Cox regression analyses with hazard ratios (HRs) and 95% confidence intervals (CIs). Subgroup analyses of age, gender, body mass index, and comorbidity were also performed.

**Results:**

The median follow‐up time was 83 months. A total of 3462 patients died for all‐cause and 1064 died due to CVD. After adjusting for covariates, we found that high adherence to AHEI‐2010 (HR = 0.84 for all‐cause; HR = 0.72 for CVD), and MED (HR = 0.84 for all‐cause; HR = 0.77 for CVD) diet were associated with decreased risks of both all‐cause and CVD‐specific mortality. In patients who aged ≥65 years old, were normal/overweight, without complications, the relationships between different dietary patterns and risk of mortality were different.

**Conclusion:**

High scores of AHEI‐2010 and MED may be associated with decreased risks of all‐cause and CVD‐specific mortality in patients with HTN.

## Graphical abstract

The role of dietary patterns in the risk of mortality in patients with HTN has been unclear. This study explored the relationship between different dietary patterns and all‐cause/CVD‐specific mortality in HTN patients. The study data were extracted from the NHANES database. After average 83 months' follow‐up, we found that high adherence to AHEI‐2010, and MED diet were associated with decreased risks of both all‐cause and CVD‐specific mortality.

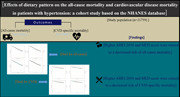

**Abbreviations**
AHA/ACC: the American Heart Association/American College of Cardiology
AHEI‐2010: the Alternate Healthy Eating Index‐2010
BMI: body mass index
Ca: calcium
CDC: the Centers for Disease Control and Prevention
CKD‐EPI: the Chronic Kidney Disease Epidemiology Collaboration
CNPP: the USDA's Center for Nutrition Policy and Promotion
COPD: chronic obstructive pulmonary disease
Cr: creatinine
CRP: C‐reactive protein
CVD: cardiovascular disease mortality
DASH: the Dietary Approaches to Stop Hypertension
DGA: the Dietary Guidelines of Americans
DHA: docosahexaenoic acid
DM: diabetes mellitus
eGFR: estimated glomerular filtration rate
EPA: timnodonic acid
FFQs: food frequency questionnaires
HB: hemoglobin
HDL‐C: high‐density lipoprotein cholesterol
HEI‐2015: the Healthy Eating Index‐2015
HIV: human immunodeficiency virus
HTN: hypertension
ICD: the International Statistical Classification of Disease.
K: potassium
LDL‐C: low‐density lipoprotein cholesterol
MCQ: multiple‐choice question
MEC: mobile examination center
MED: the Mediterranean score
MET: metabolic equivalent
Mg: magnesium
Na: sodium
NCHS: the National Center for Health Statistics
NHANES: the National Health and Nutrition Examination Survey
PIR: poverty‐to‐income ratio
PUFAs: polyunsaturated fatty acids
TG: triglyceride

## 1 INTRODUCTION

Hypertension (HTN) is a clinical syndrome characterized by elevated systolic blood pressure (SBP)/diastolic blood pressure (DBP) ≥ 140/90 mmHg, affecting 1.13 billion adults worldwide.^1^ HTN is a major modifiable risk factor for cardiovascular disease (CVD),^2^ and patients with HTN have higher risk of both all‐cause and CVD‐specific mortality compared with the general population.^3^ It is paramount to identify effective strategies to improve the prognosis of HTN individuals.

Diet adjustment, a mean of nondrug treatment, is an important part of HTN management.^4^ Previous studies have showed that dietary patterns can significantly impact a HTN patient's health, with a noticeable effect on blood pressure (BP).^5^, ^6^ A meta‐analysis of 30 randomized controlled trials found that Dietary Approaches to Stop Hypertension (DASH) significantly decreased SBP and DBP in adults with HTN.^7^ Adherence to the DASH in HTN patients has been associated with a lower risk of all‐cause mortality.^8^ A systematic review and meta‐analysis of 15 prospective cohort studies showed that higher diet quality characterized by the Alternate Healthy Eating Index (AHEI) was significantly related to decreasing all‐cause mortality.^9^ Hu et al.^10^ found that higher adherence to the Healthy Eating Index 2015 (HEI‐2015) diet was linked to lower risks of CVD‐specific and all‐cause mortality among US adults. The adoption of the Mediterranean (MED) diet was also accompanied by a relatively small, but yet significant BP reduction in HTN patients.^11^ However, studies on the association between different diet patterns and prognosis in HTN patients have been scarce.

Herein, this study aims to explore the effects of different diet patterns on the all‐cause and CVD‐specific mortality in HTN patients, in order to provide some dietary guidance for the management and prognosis improvement of these patients.

## 2 METHODS

### 2.1 Study design and participants

Data of participants in this retrospective cohort study were extracted from the National Health and Nutrition Examination Survey (NHANES) database in 2005–2018. NHANES includes publicly available 2‐year cross‐sectional surveys that focus on the health and nutrition of noninstitutionalized US residents, which is administered by the Centers for Disease Control and Prevention (CDC) and the National Center for Health Statistics (NCHS).^12^ Details including questionnaires, data sets, and related documentation of study implementation are available for online access: https://wwwn.cdc.gov/nchs/nhanes/Default.aspx.

A total of 27 618 adults who diagnosed with HTN were absorbed in this study. The exclusion criteria were (1) aged <20 years old, (2) having extremely low or high total energy intake (under 500 or over 8000 kcal/day), and (3) missing the information of dietary. After excluding those who lost to follow‐up, finally, 21 757 of the adults were eligible. Since the NHANES protocol was approved by the Institutional Review Board (IRB) of the NCHS of CDC, and the data were publicly available, no ethical approval of our IRB was required.

### 2.2 Assessment of dietary patterns

The NHANES database uses two 24‐hour recalls as the principal methodology to provide detailed quantitative food and nutrient intake assessment, and uses the food frequency questionnaires (FFQs) to provide typical or qualitative data.^13^ The FFQ was calculated in standardized portion sizes and nutrient intakes as grams per day. Participants without complete dietary data and those that reported excessively high or low values for total food or energy intake (<600 kcal/day in or more than 4200 kcal/day) were excluded.^14^

The DASH dietary pattern aims at lowering BP which focuses on high amounts of fruits, vegetables, and low‐fat dairy products. DASH score was calculated based on protein, fiber, magnesium (Mg), calcium (Ca), potassium (K), total fat, saturated fat, cholesterol, and sodium (Na) to examine the degree of adherence to the DASH. Provide one point when meeting the goal for each component, provide 0.5 points when meeting an intermediate goal of DASH and give zero points when the nutrient content of the DASH control diet and meeting neither goal. The control diet targets are from the previous DASH study.^15^ This pattern score ranges between 0 and 9 points, where higher scores indicate greater adherence to the DASH dietary pattern.

The AHEI‐2010 includes information of 11 components of food groups and nutrients that are associated consistently with lower risk of chronic diseases in clinical and epidemiologic investigations, which is based on a comprehensive review of the relevant literature and discussions with other nutrition researchers.^16^ Briefly, higher intakes of vegetables, fruits, whole grains, nuts and legumes, long‐chain ω‐3 fatty acids (timnodonic acid and docosahexaenoic acid), polyunsaturated fatty acids, and moderate alcohol count favorably, whereas higher intakes of sugar‐sweetened beverages, red/processed meats, trans fat, and Na count unfavorably. Each component of the score is rated on a scale of 0–10, and the total AHEI‐2010 score ranged from 0 (nonadherence) to 110 (perfect adherence).

The HEI‐2015 is a diet quality index to assess the adherence to the Dietary Guidelines in Americans, which were developed by the USDA's Center for Nutrition Policy and Promotion.^7^ HEI‐2015 has replaced empty calories with saturated fat and added sugar basing on the HEI‐2010. HEI‐2015 focuses on the consumption of total fruits, whole fruits, total vegetables, greens and beans, whole grains, dairy foods, total protein foods, seafood and plant proteins, fatty acids, refined grains, Na, added sugars, and saturated fats. The score ranges from 0 to 100 points, and the higher score reflects the healthier eating.

The MED score was calculated according to the information in the Food Patterns Equivalents database (developed by the US Department of Agriculture): https://www.ars.usda.gov/northeast-areaeltsvillee-md-bhnrceltsvillee-human-nutrition-research-center/food-surveys-research-group/docs/fped-databases/. In summary, the calculation was based on assessed intakes of total fruits, vegetables (except potatoes), whole grains, legumes, nuts, fish, red and processed meat, ratio of monounsaturated to saturated fat, and alcohol. A single point was assigned to participants whose intakes were greater than the median of the study cohort, except for red and processed meat and alcohol. For alcohol and red/processed meat, one point was assigned to those who had moderate alcohol consumption (10–25 g/day for men and 5–15 g/day for women) or a meat intake that was less than the median for the cohort. If these criteria were not met, participants received point of zero. Hence, higher MED (total score = 18) score represents greater adherence to the MED diet.^17^

In the current study, the four dietary patterns were divided into three levels, respectively, according to their tertiles, including HEI‐2015: ≤46.64, (46.64, 58.28), and >58.28, AHEI‐2010: ≤30.5, (30.5, 39), and >39, DASH: ≤1.5, (1.5, 2.5), and >2.5, and MED score: ≤3, (3, 5), and >5.

### 2.3 Variables collection

We collected the variables including age, gender, race, education level, marital status, poverty‐to‐income ratio (PIR), smoking status, drinking status, height, weight, body mass index (BMI), waistline, physical activity, total energy intake, estimated glomerular filtration rate (eGFR), platelet, hemoglobin (HB), creatinine (Cr), C‐reactive protein (CRP), CVD, diabetes mellitus (DM), dyslipidemia, cancer, asthma, anemia treatment, gout, chronic obstructive pulmonary disease (COPD), dialysis, hypothyroidism, human immunodeficiency virus (HIV) infection, and drug abuse from NHANES.

Physical activity was calculated by the physical activity questionnaire in NHANES, using the following formula: energy expenditure (metabolic equivalent [MET]·min) = recommended MET × exercise time of corresponding activity (min). Participants who had laboratory diagnosis of fasting plasma glucose ≥7 mmol/L, HbA1c ≥ 6.5% (48 mmol/L), self‐reported previous diagnosis of DM by medical professionals, or taking glucose‐lowering agents or insulin (survey or prescription medication check) were considered to have DM. Dyslipidemia was defined as having total cholesterol ≥ 200 mg/dL (5.2 mmol/L), or triglyceride (TG) ≥ 150 mg/dL (1.7 mmol/L), or low‐density lipoprotein cholesterol ≥ 130 mg/dL (3.4 mmol/L), or high‐density lipoprotein cholesterol ≤ 40 mg/dL (1.0 mmol/L), or self‐reported hypercholesterolemia, or use of a cholesterol medication.^10^ Medication data were compiled from the prescription drug questionnaire taken during the home interview.^11^ According to the multiple‐choice question (MCQ) in NHANES: “Have you ever been told you had (congestive) heart failure, coronary heart disease, angina/angina pectoris, heart attack and stroke” or using of cardiovascular drugs in any one or more diseases or medications was diagnosed as CVD. Renal function was calculated using the Chronic Kidney Disease Epidemiology Collaboration equation^18^: eGFR = 141 × min (SCr/κ, 1) α × max (SCrκ,1) − 1.209 × 0.993Age × 1.018 (if female) × 1.159 (if Black) where SCr (mg/dL) is serum Cr collected as part of the standard biochemistry profile, κ is 0.7 if female or 0.9 if male, α is −0.329 if female or −.411 if male, min is the minimum of SCr/κ or 1, and max is the maximum of SCr/κ or 1. Participants with SBP ≥ 130 mmHg or DBP ≥ 80 mmHg were defined as HTN according to the American Heart Association/American College of Cardiology 2017 guideline. If the BP measurement data were missing, patients with antihypertensive medication prescription in the clinical history “Are you now taking prescribed medicine” were also considered hypertensive. Survey participants who had been told by a doctor or other health care provider to have emphysema (MCQ160G) and/or chronic bronchitis (MCQ160K) were considered COPD. Subjects who were asked “Has a doctor or other health professional ever told you that you had gout?” and had positive answers were categorized into gout subjects. Information of hypothyroidism, anemia, asthma, cancer, and HIV infection were all collected through the household interviews and MCQs. NHANES collected participants' blood samples in the mobile examination center (MEC) for further examinations of clinical indicators.

### 2.4 Outcomes and follow‐up

The study outcomes were all‐cause mortality and CVD‐specific mortality. The mortality information of patients was queried using the NHANES‐linked social security account through the website: https://www.cdc.gov/nchs/data-linkage/mortality.htm. CVD‐specific mortality was defined and classified by the International Statistical Classification of Disease (ICD) codes, 10th revision (coded I00‐I78).^19^ The follow‐up ended until patients died or up to December 31, 2019.

### 2.5 Statistical analysis

Normal distributed data were described by mean ± standard deviation (mean ± SD), and *t* test was used for comparation. Data with skewed distribution were described by median and quartiles [M (Q1, Q3)], and Mann–Whitney U rank sum test was used for comparation. The composition ratio (N [%]) was used to describe the distribution of measurement data, and we used chi‐square test for comparation.

The MEC exam weight (wtmec2yr) was used for weighting since data sets in the current study incorporated seven cycles of NHANES 2‐year data collection. Weighted univariate Cox regression analysis was used to screen covariates related to all‐cause mortality and CVD‐specific mortality. Univariate and multivariate Cox regression analyses were used to explore the relationship of different dietary patterns and all‐cause mortality and CVD‐specific mortality, respectively. Model 1 was the crude model. Model 2 adjusted for demographic data including age, gender, education level, and marital status. Model 3 of all‐cause mortality adjusted for age, race, education level, marital status, PIR, physical activity, smoking, total energy intake, eGFR, drinking, BMI, DM, CVD, dyslipidemia, cancer, anemia treatment, gout, COPD, HB, dialysis, CRP, and hypothyroidism. Model 3 of CVD‐specific mortality adjusted for age, race, education level, marital status, PIR, physical activity, smoking, eGFR, drinking, DM, dyslipidemia, cancer, anemia treatment, gout, COPD, HB, dialysis, CRP, and hypothyroidism. We further drew the Kaplan–Meier (KM) curves to reflect the influences of different dietary patterns on all‐cause mortality and CVD‐specific mortality, respectively. Subgroup analyses of age, BMI, CVD, DM, and dyslipidemia were also performed. The evaluation indexes were hazard ratios (HRs) and 95% confidence intervals (CIs). Two‐sided *p* < .05 was considered as significant.

Statistical analyses were completed by SAS 9.4 (SAS Institute.), and R version 4.2.0 (Institute for Statistics and Mathematics). Missing data were interpolated. We further performed the sensitivity analysis on characteristics of participants before and after the interpolation of missing data (Supporting Information: Table S1).

## 3 RESULTS

### 3.1 Characteristics of participants

A total of 27 618 patients with HTN were initially included. We excluded those who aged less than 20 years old (*n* = 662), missing dietary data (*n* = 2255), and having extreme energy intakes (<500 or >8000 kcal) (*n* = 147). Then, individuals who lost to the follow‐up were also excluded (*n* = 2795). Finally, 21 757 of the patients were eligible.

Table 1 showed the characteristics of patients with HTN. After a median 83 months' follow‐up, 3 462 patients died for all‐cause while that 1064 died due to CVD. The average age of eligible patients was 53.45 years old and 53.04% of them were male. We found the HEI‐2015, AHEI‐2010, DASH, and MED scores were all significantly different between survival group and all‐cause mortality group, and between the survival group and CVD‐specific mortality group (all *p* < .05).

**Table 1:** Characteristics of patients with HTN.

Variables	Total (*n* = 21 757)	All‐cause mortality		*p* Value	CVD‐specific mortality		*p* Value
		Survival (*n* = 18 295)	Mortality (*n* = 3462)		Non‐CVD‐specific mortality (*n* = 20 693)	CVD‐specific mortality (*n* = 1064)	
Age, years, Mean ± SD	53.45 ± 16.05	50.81 ± 15.10	67.43 ± 13.53	<.001	52.63 ± 15.76	69.50 ± 13.04	<.001
Gender, *n* (%)				<.001			.106
Male	11 540 (53.04)	9590 (52.42)	1950 (56.33)		10 950 (52.92)	590 (55.45)	
Female	10 217 (46.96)	8705 (47.58)	1512 (43.67)		9743 (47.08)	474 (44.55)	
Race, *n* (%)				<.001			<.001
Mexican American	3068 (14.10)	2795 (15.28)	273 (7.89)		3003 (14.51)	65 (6.11)	
Other Hispanic	1561 (7.17)	1408 (7.70)	153 (4.42)		1502 (7.26)	59 (5.55)	
Non‐Hispanic White	9946 (45.71)	7885 (43.10)	2061 (59.53)		9332 (45.10)	614 (57.71)	
Non‐Hispanic Black	5687 (26.14)	4832 (26.41)	855 (24.70)		5393 (26.06)	294 (27.63)	
Other race—including multiracial	1495 (6.87)	1375 (7.52)	120 (3.47)		1463 (7.07)	32 (3.01)	
Education level, *n* (%)				<.001			<.001
<9th grade	2413 (11.09)	1862 (10.18)	551 (15.92)		2245 (10.85)	168 (15.79)	
9–11th grade	3194 (14.68)	2523 (13.79)	671 (19.38)		2981 (14.41)	213 (20.02)	
High school Grad/GED or equivalent	5343 (24.56)	4397 (24.03)	946 (27.33)		5047 (24.39)	296 (27.82)	
Some college or AA degree	6470 (29.74)	5612 (30.68)	858 (24.78)		6213 (30.02)	257 (24.15)	
College graduate or above	4337 (19.93)	3901 (21.32)	436 (12.59)		4207 (20.33)	130 (12.22)	
Marital status, *n* (%)				<.001			<.001
Married	11 690 (53.73)	10 138 (55.41)	1552 (44.83)		11 230 (54.27)	460 (43.23)	
Widowed	2074 (9.53)	1198 (6.55)	876 (25.30)		1764 (8.52)	310 (29.14)	
Divorced	2806 (12.90)	2310 (12.63)	496 (14.33)		2672 (12.91)	134 (12.59)	
Separated	824 (3.79)	706 (3.86)	118 (3.41)		787 (3.80)	37 (3.48)	
Never married	2898 (13.32)	2631 (14.38)	267 (7.71)		2812 (13.59)	86 (8.08)	
Living with partner	1465 (6.73)	1312 (7.17)	153 (4.42)		1428 (6.90)	37 (3.48)	
PIR, *n* (%)				<.001			.097
≤1	3920 (18.02)	3185 (17.41)	735 (21.23)		3708 (17.92)	212 (19.92)	
>1	17 837 (81.98)	15 110 (82.59)	2727 (78.77)		16 985 (82.08)	852 (80.08)	
Smoking, *n* (%)				<.001			<.001
Yes	10 588 (48.66)	8460 (46.24)	2128 (61.47)		10 001 (48.33)	587 (55.17)	
No	11 169 (51.34)	9835 (53.76)	1334 (38.53)		10 692 (51.67)	477 (44.83)	
Drinking, *n* (%)				<.001			<.001
Yes	14 547 (66.86)	12 352 (67.52)	2195 (63.40)		13 906 (67.20)	641 (60.24)	
No	7210 (33.14)	5943 (32.48)	1267 (36.60)		6787 (32.80)	423 (39.76)	
Height, cm, Mean ± SD	168.06 ± 10.43	168.32 ± 10.41	166.65 ± 10.39	<.001	168.17 ± 10.43	165.76 ± 10.12	<.001
Weight, kg, Mean ± SD	86.99 ± 23.67	87.79 ± 23.54	82.62 ± 23.94	<.001	87.20 ± 23.69	82.78 ± 23.01	<.001
BMI, kg/m^2^, Mean ± SD	30.71 ± 7.50	30.91 ± 7.49	29.66 ± 7.48	<.001	30.75 ± 7.52	29.98 ± 7.22	.001
Waistline, cm, Mean ± SD	103.64 ± 16.57	103.60 ± 16.50	103.83 ± 16.92	.455	103.61 ± 16.58	104.29 ± 16.33	.191
Physical activity, MET*min, M (Q_1_, Q_3_)	180.00 (0.00, 735.00)	240.00 (0.00, 900.00)	0.00 (0.00, 270.00)	<.001	200.00 (0.00, 800.00)	0.00 (0.00, 240.00)	<.001
Total energy intake, Kcal, M (Q_1_, Q_3_)	1917.00 (1448.00, 2492.00)	1961.50 (1483.00, 2550.00)	1704.50 (1307.50, 2189.00)	<.001	1934.50 (1463.50, 2514.00)	1615.25 (1291.50, 2108.75)	<.001
eGFR, mL/min/1.73*m^2^, Mean ± SD	90.63 ± 22.11	93.65 ± 20.50	74.72 ± 23.48	<.001	91.62 ± 21.63	71.47 ± 22.67	<.001
Platelet, K/uL, Mean ± SD	8.18 ± 0.92	8.20 ± 0.91	8.09 ± 0.97	<.001	8.18 ± 0.92	8.09 ± 1.01	.005
HB, g/dL, Mean ± SD	14.19 ± 1.62	14.26 ± 1.59	13.82 ± 1.75	<.001	14.22 ± 1.61	13.67 ± 1.71	<.001
Cr, umol/L, M (Q_1_, Q_3_)	0.90 (0.77, 1.08)	0.90 (0.75, 1.04)	1.00 (0.81, 1.22)	<.001	0.90 (0.76, 1.06)	1.02 (0.84, 1.30)	<.001
CRP, mg/dL, *n* (%)				.061			.096
≤0.3	7432 (34.16)	6250 (34.16)	1182 (34.14)		7044 (34.04)	388 (36.47)	
>0.3	8331 (38.29)	6954 (38.01)	1377 (39.77)		7956 (38.45)	375 (35.24)	
Unknown	5994 (27.55)	5091 (27.83)	903 (26.08)		5693 (27.51)	301 (28.29)	
HEI‐2015, Mean ± SD	53.22 ± 13.20	53.10 ± 13.23	53.83 ± 13.03	.003	53.15 ± 13.19	54.53 ± 13.32	<.001
AHEI‐2010, Mean ± SD	35.57 ± 9.67	35.51 ± 9.79	35.84 ± 9.02	.054	35.56 ± 9.71	35.58 ± 8.92	.948
DASH, M (Q_1_, Q_3_)	2.00 (1.00, 3.00)	2.00 (1.00, 3.00)	2.00 (1.00, 3.00)	.024	2.00 (1.00, 3.00)	2.00 (1.00, 3.50)	<.001
MED score, M (Q_1_, Q_3_)	4.00 (3.00, 6.00)	4.00 (3.00, 6.00)	4.00 (2.00, 5.00)	<.001	4.00 (3.00, 6.00)	4.00 (3.00, 5.00)	.071
HEI‐2015, *n* (%)				<.001			.003
≤46.64	7187 (33.03)	6137 (33.54)	1050 (30.33)		6879 (33.24)	308 (28.95)	
(46.64, 58.28)	7173 (32.97)	6017 (32.89)	1156 (33.39)		6823 (32.97)	350 (32.89)	
>58.28	7397 (34.00)	6141 (33.57)	1256 (36.28)		6991 (33.78)	406 (38.16)	
AHEI‐2010, *n* (%)				.007			.017
≤30.5	6646 (30.55)	5655 (30.91)	991 (28.63)		6352 (30.70)	294 (27.63)	
(30.5, 39)	6903 (31.73)	5737 (31.36)	1166 (33.68)		6526 (31.54)	377 (35.43)	
>39	8208 (37.73)	6903 (37.73)	1305 (37.69)		7815 (37.77)	393 (36.94)	
DASH, *n* (%)				.821			.073
≤1.5	6486 (29.81)	5469 (29.89)	1017 (29.38)		6202 (29.97)	284 (26.69)	
(1.5, 2.5)	2802 (12.88)	2356 (12.88)	446 (12.88)		2657 (12.84)	145 (13.63)	
>2.5	12 469 (57.31)	10 470 (57.23)	1999 (57.74)		11 834 (57.19)	635 (59.68)	
MED score, *n* (%)				<.001			.008
≤3	5325 (24.47)	4459 (24.37)	866 (25.01)		5074 (24.52)	251 (23.59)	
(3, 5)	4155 (19.10)	3399 (18.58)	756 (21.84)		3913 (18.91)	242 (22.74)	
>5	12 277 (56.43)	10 437 (57.05)	1840 (53.15)		11 706 (56.57)	571 (53.67)	
CVD, *n* (%)				<.001			<.001
No	17 833 (81.96)	15 857 (86.67)	1976 (57.08)		17 317 (83.69)	516 (48.50)	
Yes	3924 (18.04)	2438 (13.33)	1486 (42.92)		3376 (16.31)	548 (51.50)	
DM, n (%)				<.001			<.001
No	16 482 (75.75)	14 310 (78.22)	2172 (62.74)		15 822 (76.46)	660 (62.03)	
Yes	5275 (24.25)	3985 (21.78)	1290 (37.26)		4871 (23.54)	404 (37.97)	
Dyslipidemia, *n* (%)				<.001			<.001
No	5118 (23.52)	4424 (24.18)	694 (20.05)		4925 (23.80)	193 (18.14)	
Yes	16 639 (76.48)	13 871 (75.82)	2768 (79.95)		15 768 (76.20)	871 (81.86)	
Cancer, *n* (%)				<.001			<.001
No	19 373 (89.04)	16 675 (91.15)	2698 (77.93)		18 511 (89.46)	862 (81.02)	
Yes	2384 (10.96)	1620 (8.85)	764 (22.07)		2182 (10.54)	202 (18.98)	
Asthma, *n* (%)				.907			.414
No	18 500 (85.03)	15 554 (85.02)	2946 (85.10)		17 586 (84.99)	914 (85.90)	
Yes	3257 (14.97)	2741 (14.98)	516 (14.90)		3107 (15.01)	150 (14.10)	
Anemia treatment, *n* (%)				<.001			<.001
No	20 765 (95.51)	17 613 (96.32)	3152 (91.20)		19 813 (95.81)	952 (89.73)	
Yes	976 (4.49)	672 (3.68)	304 (8.80)		867 (4.19)	109 (10.27)	
Gout, *n* (%)				<.001			<.001
No	12 797 (58.82)	10 822 (59.15)	1975 (57.05)		12 196 (58.94)	601 (56.48)	
Yes	1013 (4.66)	705 (3.85)	308 (8.90)		900 (4.35)	113 (10.62)	
Unknown	7947 (36.53)	6768 (36.99)	1179 (34.06)		7597 (36.71)	350 (32.89)	
COPD, *n* (%)				<.001			<.001
No	5081 (23.35)	4672 (25.54)	409 (11.81)		4943 (23.89)	138 (12.97)	
Yes	266 (1.22)	191 (1.04)	75 (2.17)		247 (1.19)	19 (1.79)	
Unknown	16 410 (75.42)	13 432 (73.42)	2978 (86.02)		15 503 (74.92)	907 (85.24)	
Dialysis, *n* (%)				<.001			<.001
No	844 (3.88)	578 (3.16)	266 (7.68)		761 (3.68)	83 (7.80)	
Yes	112 (0.51)	46 (0.25)	66 (1.91)		96 (0.46)	16 (1.50)	
Unknown	20 801 (95.61)	17 671 (96.59)	3130 (90.41)		19 836 (95.86)	965 (90.70)	
Hypothyroidism, *n* (%)				<.001			<.001
No	4266 (19.61)	3315 (18.12)	951 (27.47)		3965 (19.16)	301 (28.29)	
Yes	132 (0.61)	84 (0.46)	48 (1.39)		117 (0.57)	15 (1.41)	
Unknown	17 359 (79.79)	14 896 (81.42)	2463 (71.14)		16 611 (80.27)	748 (70.30)	
HIV infection, *n* (%)				<.001			<.001
No	10 512 (48.32)	10 005 (54.69)	507 (14.64)		10 380 (50.16)	132 (12.41)	
Yes	66 (0.30)	61 (0.33)	5 (0.14)		66 (0.32)	0 (0.00)	
Unknown	11 179 (51.38)	8229 (44.98)	2950 (85.21)		10 247 (49.52)	932 (87.59)	
Drug abuse, *n* (%)				<.001			<.001
No	4860 (22.34)	4488 (24.53)	372 (10.75)		4794 (23.17)	66 (6.20)	
Yes	1464 (6.73)	1272 (6.95)	192 (5.55)		1398 (6.76)	66 (6.20)	
Unknown	15 433 (70.93)	12 535 (68.52)	2898 (83.71)		14 501 (70.08)	932 (87.59)	

Abbreviations: AA degree, associate degree; AHEI, Alternate Healthy Eating Index; BMI, body mass index; COPD, chronic obstructive pulmonary disease; Cr, creatinine; CRP, C‐reactive protein; CVD, cardiovascular disease; DASH, dietary approaches to stop hypertension; DM, diabetes mellitus; eGFR, estimated glomerular filtration rate; GED, general equivalency diploma; HB, hemoglobin; HEI, Healthy Eating Index; HIV, human immunodeficiency virus; HTN, hypertension; M, medium; MED, Mediterranean; MET, metabolic equivalent; PIR, poverty‐to‐income ratio; Q1, first quartile; Q3, third quartile; SD, standard deviation.

### 3.2 Association between different dietary patterns and all‐cause mortality and CVD‐specific mortality in patients with HTN

Covariates respectively related to all‐cause mortality and CVD‐specific mortality were showed in Supporting Information: Table S2. We found that age, race, education level, marital status, PIR, physical activity, total energy intake, smoking, eGFR, drinking, DM, CVD, dyslipidemia, cancer, asthma, anemia treatment, gout, COPD, HB, and dialysis were both associated with all‐cause mortality and CVD‐specific mortality (all *p* < .05).

**Figure 1:**
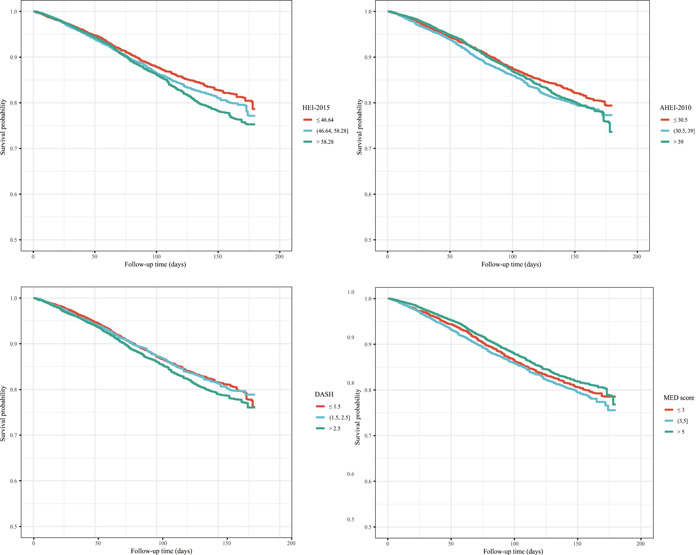
Kaplan–Meier curve of the effects of different dietary patterns on all‐cause mortality in patients with hypertension.

**Figure 2:**
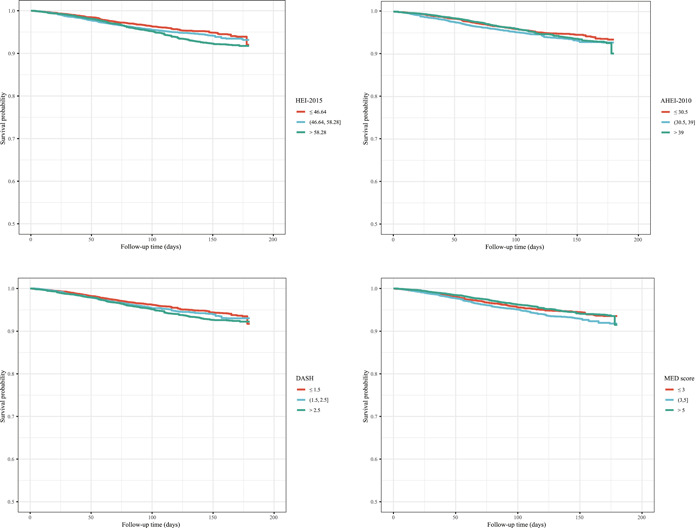
Kaplan–Meier curve of the effects of different dietary patterns on cardiovascular disease‐specific mortality in patients with hypertension.

Table 2 shows the association between different dietary patterns and all‐cause mortality and CVD‐specific mortality. After adjusting for covariates, higher AHEI‐2010 (HR = 0.84, 95% CI: [0.71–0.99]) and MED score (HR = 0.84, 95% CI: [0.71–0.99]) were related to a decreased risk of all‐cause mortality. Patients who had higher AHEI‐2010 (HR = 0.72, 95% CI: [0.56–0.93]) and MED score (HR = 0.77, 95% CI: [0.62–0.97]) were seemed to have a lower risk of CVD‐specific mortality. In addition, Figures 1 and 2 were the KM curves that reflected the effects of different level of dietary pattern scores on the all‐cause mortality and CVD‐specific mortality in patients with HTN.

**Table 2:** Association between different dietary patterns and all‐cause mortality and CVD‐specific mortality.

Variables	Model 1		Model 2		Model 3	
	HR (95% CI)	*p* Value	HR (95% CI)	*p* Value	HR (95% CI)	*p* Value
All‐cause mortality						
HEI‐2015						
≤46.64	Ref		Ref		Ref	
(46.64, 58.28)	1.13 (0.97–1.31)	.126	0.86 (0.73–1.03)	.096	0.95 (0.81–1.10)	.478
>58.28	1.39 (1.18–1.64)	<.001	0.80 (0.68–0.95)	.012	0.91 (0.76–1.08)	.257
AHEI‐2010						
≤30.5	Ref		Ref		Ref	
(30.5, 39)	1.24 (1.02–1.49)	.028	1.02 (0.85–1.22)	.844	1.04 (0.86–1.27)	.676
>39	1.17 (0.96–1.42)	.119	0.80 (0.67–0.95)	.013	0.84 (0.71–0.99)	.035
DASH						
≤1.5	Ref		Ref		Ref	
(1.5, 2.5)	1.00 (0.78–1.27)	.987	0.93 (0.74–1.16)	.507	0.97 (0.79–1.18)	.748
>2.5	1.23 (1.00–1.50)	.048	0.90 (0.76–1.07)	.222	0.98 (0.82–1.17)	.803
MED score						
≤3	Ref		Ref		Ref	
(3, 5)	1.06 (0.90–1.25)	.498	0.90 (0.78–1.04)	.152	0.97 (0.83–1.14)	.753
>5	0.91 (0.77–1.08)	.285	0.75 (0.65–0.88)	<.001	0.84 (0.71–0.99)	.036
CVD‐specific mortality						
HEI‐2015						
≤46.64	Ref		Ref		Ref	
(46.64, 58.28)	1.14 (0.88–1.47)	.333	0.81 (0.62–1.05)	.106	0.86 (0.65–1.16)	.325
>58.28	1.61 (1.20–2.16)	.001	0.80 (0.57–1.11)	.180	0.88 (0.61–1.26)	.481
AHEI‐2010						
≤30.5	Ref		Ref		Ref	
(30.5, 39)	1.20 (1.06–1.36)	.003	0.85 (0.68–1.07)	.174	0.87 (0.69–1.10)	.237
>39	0.99 (0.86–1.15)	.916	0.65 (0.51–0.84)	.001	0.72 (0.56–0.93)	.011
DASH						
≤1.5	Ref		Ref		Ref	
(1.5, 2.5)	1.03 (0.90–1.18)	.688	1.00 (0.77–1.31)	.988	1.03 (0.80–1.33)	.827
>2.5	1.13 (0.97–1.32)	.125	0.86 (0.70–1.06)	.161	0.91 (0.73–1.12)	.370
MED score						
≤3	Ref		Ref		Ref	
(3, 5)	1.17 (1.02–1.34)	.024	0.79 (0.63–0.99)	.038	0.80 (0.64–0.99)	.041
>5	1.00 (0.88–1.13)	.955	0.71 (0.57–0.88)	.002	0.77 (0.62–0.97)	.025

Abbreviations: AHEI, Alternate Healthy Eating Index; BMI, body mass index; CI, confidence interval; COPD, chronic obstructive pulmonary disease; CRP, C‐reactive protein; CVD, cardiovascular disease; DASH, Dietary Approaches to Stop Hypertension; DM, diabetes mellitus; eGFR, estimated glomerular filtration rate; HB, hemoglobin; HEI, Healthy Eating Index; HR, hazard ratio; MED, Mediterranean; PIR, poverty‐to‐income ratio; Ref, reference.

Model 1: Crude model.

Model 2: Adjusted for age, gender, education level, and marital status.

Model 3 of all‐cause mortality: Adjusted for age, race, education level, marital status, PIR, physical activity, smoking, total energy intake, eGFR, drinking, BMI, DM, CVD, dyslipidemia, cancer, anemia treatment, gout, COPD, HB, dialysis, CRP, and hypothyroidism.

Model 3 of CVD‐specific mortality: Adjusted for age, race, education level, marital status, PIR, physical activity, smoking, eGFR, drinking, DM, dyslipidemia, cancer, anemia treatment, gout, COPD, HB, dialysis, CRP, and hypothyroidism.

### 3.3 Subgroup analyses of age, BMI, gender, CVD, DM, and dyslipidemia

Table 3 shows the results of subgroup analyses. In patients aged ≥65 years old, high HEI‐2015, AHEI‐2010, and MED scores were all associated with decreased risks of both all‐cause mortality and CVD‐specific mortality (all *p* < .05). Additionally, high DASH score was only linked to a decreased risk of all‐cause mortality (HR = 0.84, 95% CI: [0.72–0.99]).

**Table 3:** Association between different dietary patterns and all‐cause and CVD‐specific mortality in age, gender, BMI, and comorbidity subgroups.

Subgroups	Dietary patterns	All‐cause mortality		CVD‐specific mortality	
		HR (95% CI)	*p* Value	HR (95% CI)	*p* Value
Age <65	HEI‐2015				
	≤46.64	Ref		Ref	
	(46.64, 58.28)	0.98 (0.78–1.23)	.837	0.96 (0.77–1.21)	.744
	>58.28	1.28 (0.90–1.82)	.164	1.31 (0.93–1.84)	.124
	AHEI‐2010				
	≤30.5	Ref		Ref	
	(30.5, 39)	1.15 (0.80–1.67)	.451	1.19 (0.81–1.75)	.379
	>39	0.97 (0.68–1.39)	.883	1.01 (0.71–1.42)	.965
	DASH				
	≤1.5	Ref		Ref	
	(1.5, 2.5)	0.78 (0.52–1.18)	.240	0.82 (0.55–1.23)	.345
	>2.5	1.02 (0.70–1.49)	.904	1.13 (0.78–1.62)	.515
	MED score				
	≤3	Ref		Ref	
	(3, 5)	1.08 (0.81–1.44)	.610	1.09 (0.81–1.46)	.582
	>5	0.87 (0.63–1.20)	.404	0.87 (0.63–1.20)	.399
Age ≥65	HEI‐2015				
	≤46.64	Ref		Ref	
	(46.64, 58.28)	0.93 (0.81–1.08)	.357	0.91 (0.71–1.17)	.465
	>58.28	0.73 (0.63–0.85)	<.001	0.73 (0.56–0.97)	.028
	AHEI‐2010				
	≤30.5	Ref		Ref	
	(30.5, 39)	0.90 (0.76–1.05)	.182	0.75 (0.59–0.96)	.021
	>39	0.73 (0.63–0.85)	<.001	0.66 (0.52–0.84)	<.001
	DASH				
	≤1.5	Ref		Ref	
	(1.5, 2.5)	1.11 (0.94–1.30)	.223	1.22 (0.93–1.60)	.155
	>2.5	0.84 (0.72–0.99)	.034	0.90 (0.69–1.18)	.453
	MED score				
	≤3	Ref		Ref	
	(3, 5)	0.89 (0.77–1.02)	.101	0.88 (0.73–1.05)	.162
	>5	0.80 (0.69–0.91)	.001	0.74 (0.60–0.92)	.006
Normal/overweight	HEI‐2015				
	≤46.64	Ref		Ref	
	(46.64, 58.28)	0.85 (0.67–1.07)	.174	0.91 (0.62–1.31)	.603
	>58.28	0.81 (0.63–1.03)	.088	0.97 (0.60–1.58)	.910
	AHEI‐2010				
	≤30.5	Ref		Ref	
	(30.5, 39)	0.89 (0.73–1.08)	.248	0.88 (0.67–1.15)	.341
	>39	0.74 (0.58–0.94)	.014	0.73 (0.54–0.99)	.043
	DASH				
	≤1.5	Ref		Ref	
	(1.5, 2.5)	0.92 (0.72–1.18)	.525	1.28 (0.98–1.67)	.065
	>2.5	0.82 (0.62–1.09)	.173	1.10 (0.72–1.69)	.659
	MED score				
	≤3	Ref		Ref	
	(3, 5)	1.09 (0.89–1.34)	.393	1.31 (0.93–1.84)	.120
	>5	0.74 (0.62–0.88)	<.001	0.78 (0.57–1.07)	.130
Obesity	HEI‐2015				
	≤46.64	Ref		Ref	
	(46.64, 58.28)	1.12 (0.91–1.40)	.289	0.90 (0.60–1.34)	.594
	>58.28	1.03 (0.78–1.37)	.823	0.82 (0.53–1.25)	.350
	AHEI‐2010				
	≤30.5	Ref		Ref	
	(30.5, 39)	1.19 (0.89–1.61)	.242	1.17 (0.67–2.03)	.583
	>39	0.89 (0.68–1.16)	.398	0.70 (0.44–1.12)	.135
	DASH				
	≤1.5	Ref		Ref	
	(1.5, 2.5)	0.98 (0.75–1.29)	.911	0.82 (0.57–1.18)	.281
	>2.5	1.05 (0.80–1.37)	.741	1.01 (0.68–1.49)	.977
	MED score				
	≤3	Ref		Ref	
	(3, 5)	0.80 (0.61–1.06)	.126	0.88 (0.60–1.28)	.501
	>5	1.00 (0.77–1.29)	.970	1.10 (0.77–1.58)	.587
Male	HEI‐2015				
	≤46.64	Ref		Ref	
	(46.64, 58.28)	1.07 (0.81–1.43)	.623	0.93 (0.66–1.29)	.652
	>58.28	1.04 (0.79–1.35)	.798	1.13 (0.74–1.72)	.571
	AHEI‐2010				
	≤30.5	Ref		Ref	
	(30.5, 39)	0.98 (0.76–1.26)	.861	0.98 (0.59–1.62)	.934
	>39	0.87 (0.73–1.04)	.130	0.88 (0.58–1.32)	.538
	DASH				
	≤1.5	Ref		Ref	
	(1.5, 2.5)	0.99 (0.75–1.32)	.950	0.99 (0.68–1.43)	.945
	>2.5	1.03 (0.81–1.32)	.815	1.12 (0.73–1.71)	.595
	MED score				
	≤3	Ref		Ref	
	(3, 5)	1.08 (0.83–1.39)	.567	1.22 (0.87–1.70)	.247
	>5	0.87 (0.65–1.17)	.360	1.13 (0.80–1.60)	.480
Female	HEI‐2015				
	≤46.64	Ref		Ref	
	(46.64, 58.28)	0.85 (0.66–1.08)	.187	0.89 (0.57–1.37)	.590
	>58.28	0.82 (0.59–1.13)	.227	0.76 (0.48–1.20)	.240
	AHEI‐2010				
	≤30.5	Ref		Ref	
	(30.5, 39)	1.17 (0.98–1.41)	.090	1.02 (0.74–1.40)	.895
	>39	0.84 (0.67–1.05)	.122	0.78 (0.53–1.15)	.215
	DASH				
	≤1.5	Ref		Ref	
	(1.5, 2.5)	0.95 (0.73–1.23)	.691	1.23 (0.92–1.65)	.159
	>2.5	0.89 (0.71–1.11)	.307	0.94 (0.64–1.39)	.765
	MED score				
	≤3	Ref		Ref	
	(3, 5)	0.83 (0.62–1.12)	.224	1.12 (0.77–1.64)	.545
	>5	0.79 (0.62–1.02)	.069	0.83 (0.59–1.17)	.293
CVD	HEI‐2015				
	≤46.64	Ref		Ref	
	(46.64, 58.28)	1.17 (0.91–1.51)	.229	0.98 (0.67–1.42)	.906
	>58.28	1.12 (0.85–1.48)	.406	1.18 (0.77–1.82)	.450
	AHEI‐2010				
	≤30.5	Ref		Ref	
	(30.5, 39)	1.10 (0.82–1.47)	.518	0.81 (0.54–1.22)	.321
	>39	0.98 (0.75–1.27)	.865	0.59 (0.40–0.87)	.008
	DASH				
	≤1.5	Ref		Ref	
	(1.5, 2.5)	0.87 (0.63–1.20)	.385	1.04 (0.71–1.54)	.833
	>2.5	0.82 (0.62–1.09)	.177	1.05 (0.66–1.66)	.843
	MED score				
	≤3	Ref		Ref	
	(3, 5)	0.86 (0.65–1.14)	.291	1.18 (0.75–1.87)	.473
	>5	0.78 (0.59–1.03)	.084	0.84 (0.59–1.20)	.346
Non‐CVD	HEI‐2015				
	≤46.64	Ref		Ref	
	(46.64, 58.28)	0.85 (0.68–1.07)	.167	0.79 (0.55–1.13)	.192
	>58.28	0.81 (0.67–0.98)	.032	0.71 (0.44–1.13)	.152
	AHEI‐2010				
	≤30.5	Ref		Ref	
	(30.5, 39)	0.98 (0.79–1.22)	.871	1.02 (0.73–1.42)	.916
	>39	0.77 (0.63–0.94)	.009	0.93 (0.66–1.31)	.678
	DASH				
	≤1.5	Ref		Ref	
	(1.5, 2.5)	0.94 (0.73–1.22)	.655	0.98 (0.64–1.50)	.923
	>2.5	0.96 (0.80–1.15)	.639	0.97 (0.65–1.44)	.866
	MED score				
	≤3	Ref		Ref	
	(3, 5)	1.06 (0.83–1.35)	.635	1.12 (0.82–1.53)	.475
	>5	0.87 (0.69–1.10)	.259	1.02 (0.75–1.39)	.904
DM	HEI‐2015				
	≤46.64	Ref		Ref	
	(46.64, 58.28)	0.94 (0.66–1.34)	.749	0.73 (0.48–1.11)	.146
	>58.28	0.95 (0.69–1.29)	.725	0.82 (0.53–1.29)	.400
	AHEI‐2010				
	≤30.5	Ref		Ref	
	(30.5, 39)	1.01 (0.77–1.32)	.953	0.97 (0.65–1.44)	.881
	>39	0.95 (0.76–1.18)	.644	0.87 (0.54–1.41)	.569
	DASH				
	≤1.5	Ref		Ref	
	(1.5, 2.5)	0.99 (0.75–1.32)	.972	1.05 (0.69–1.60)	.834
	>2.5	0.99 (0.76–1.29)	.932	1.13 (0.66–1.91)	.661
	MED score				
	≤3	Ref		Ref	
	(3, 5)	0.92 (0.75–1.14)	.458	1.02 (0.61–1.69)	.944
	>5	0.85 (0.69–1.04)	.108	0.92 (0.64–1.33)	.664
Non‐DM	HEI‐2015				
	≤46.64	Ref		Ref	
	(46.64, 58.28)	0.94 (0.78–1.14)	.527	0.94 (0.61–1.43)	.758
	>58.28	0.86 (0.70–1.07)	.177	0.91 (0.53–1.58)	.739
	AHEI‐2010				
	≤30.5	Ref		Ref	
	(30.5, 39)	1.03 (0.82–1.29)	.811	0.96 (0.67–1.38)	.836
	>39	0.75 (0.60–0.95)	.015	0.72 (0.53–0.99)	.041
	DASH				
	≤1.5	Ref		Ref	
	(1.5, 2.5)	0.93 (0.73–1.19)	.572	1.06 (0.77–1.45)	.727
	>2.5	0.90 (0.70–1.15)	.400	0.96 (0.66–1.39)	.827
	MED score				
	≤3	Ref		Ref	
	(3, 5)	0.99 (0.81–1.20)	.905	1.18 (0.86–1.61)	.303
	>5	0.82 (0.65–1.03)	.090	0.98 (0.69–1.39)	.918
Dyslipidemia	HEI‐2015				
	≤46.64	Ref		Ref	
	(46.64, 58.28)	0.97 (0.79–1.20)	.797	0.82 (0.59–1.13)	.218
	>58.28	0.89 (0.71–1.10)	.287	0.79 (0.55–1.13)	.192
	AHEI‐2010				
	≤30.5	Ref		Ref	
	(30.5, 39)	1.03 (0.83–1.28)	.775	0.95 (0.71–1.28)	.756
	>39	0.83 (0.69–1.00)	.044	0.73 (0.55–0.98)	.034
	DASH				
	≤1.5	Ref		Ref	
	(1.5, 2.5)	0.98 (0.79–1.21)	.838	0.95 (0.71–1.28)	.756
	>2.5	0.95 (0.79–1.15)	.610	0.88 (0.63–1.21)	.425
	MED score				
	≤3	Ref		Ref	
	(3, 5)	0.91 (0.76–1.08)	.282	1.01 (0.79–1.29)	.929
	>5	0.82 (0.67–0.99)	.044	0.86 (0.65–1.13)	.269
Nondyslipidemia	HEI‐2015				
	≤46.64	Ref		Ref	
	(46.64, 58.28)	0.84 (0.53–1.32)	.443	1.26 (0.48–3.31)	.639
	>58.28	1.01 (0.62–1.64)	.982	1.84 (0.57–5.90)	.305
	AHEI‐2010				
	≤30.5	Ref		Ref	
	(30.5, 39)	1.08 (0.66–1.79)	.757	1.14 (0.47–2.74)	.775
	>39	0.89 (0.62–1.27)	.512	1.32 (0.70–2.50)	.397
	DASH				
	≤1.5	Ref		Ref	
	(1.5, 2.5)	0.90 (0.62–1.31)	.581	2.06 (0.99–4.29)	.052
	>2.5	0.86 (0.50–1.49)	.593	2.30 (0.81–6.56)	.119
	MED score				
	≤3	Ref		Ref	
	(3, 5)	1.28 (0.77–2.11)	.341	2.91 (1.12–7.52)	.028
	>5	0.98 (0.65–1.50)	.943	2.66 (1.02–6.94)	.046

Abbreviations: AHEI, Alternate Healthy Eating Index; BMI, body mass index; CI, confidence interval; COPD, chronic obstructive pulmonary disease; CRP, C‐reactive protein; CVD, cardiovascular disease; DASH, Dietary Approaches to Stop Hypertension; DM, diabetes mellitus; eGFR, estimated glomerular filtration rate; HB, hemoglobin; HEI, Healthy Eating Index; HR, hazard ratio; MED, Mediterranean; PIR, poverty‐to‐income ratio; Ref, reference.

*Age subgroup*: Adjusted for race, education level, marital status, PIR, physical activity, smoking, total energy intake, eGFR, drinking, BMI, DM, CVD, dyslipidemia, cancer, anemia treatment, gout, COPD, HB, dialysis, CRP, and hypothyroidism for all‐cause mortality.

Adjusted for race, education level, marital status, PIR, physical activity, smoking, eGFR, drinking, DM, dyslipidemia, cancer, anemia treatment, gout, COPD, HB, dialysis, CRP, and hypothyroidism for CVD‐specific mortality.

*BMI subgroup*: Adjusted for age, race, education level, marital status, PIR, physical activity, smoking, total energy intake, eGFR, drinking, DM, CVD, dyslipidemia, cancer, anemia treatment, gout, COPD, HB, dialysis, CRP, and hypothyroidism for all‐cause mortality.

Adjusted for age, race, education level, marital status, PIR, physical activity, smoking, eGFR, drinking, DM, dyslipidemia, cancer, anemia treatment, gout, COPD, HB, dialysis, CRP, and hypothyroidism for CVD‐specific mortality.

*Gender subgroup*: Adjusted for age, race, education level, marital status, PIR, physical activity, smoking, total energy intake, eGFR, drinking, BMI, DM, CVD, dyslipidemia, cancer, anemia treatment, gout, COPD, HB, dialysis, CRP, and hypothyroidism for all‐cause mortality.

Adjusted for age, race, education level, marital status, PIR, physical activity, smoking, eGFR, drinking, DM, dyslipidemia, cancer, anemia treatment, gout, COPD, HB, dialysis, CRP, and hypothyroidism for CVD‐specific mortality.

*CVD subgroup*: Adjusted for age, race, education level, marital status, PIR, physical activity, smoking, total energy intake, eGFR, drinking, BMI, DM, dyslipidemia, cancer, anemia treatment, gout, COPD, HB, dialysis, CRP, and hypothyroidism for all‐cause mortality.

Adjusted for age, race, education level, marital status, PIR, physical activity, smoking, eGFR, drinking, DM, dyslipidemia, cancer, anemia treatment, gout, COPD, HB, dialysis, CRP, and hypothyroidism for CVD‐specific mortality.

*DM subgroup*: Adjusted for age, race, education level, marital status, PIR, physical activity, smoking, total energy intake, eGFR, drinking, BMI, CVD, dyslipidemia, cancer, anemia treatment, gout, COPD, HB, dialysis, CRP, and hypothyroidism for all‐cause mortality.

Adjusted for age, race, education level, marital status, PIR, physical activity, smoking, eGFR, drinking, dyslipidemia, cancer, anemia treatment, gout, COPD, HB, dialysis, CRP, and hypothyroidism for CVD‐specific mortality.

*Dyslipidemia subgroup*: Adjusted for age, race, education level, marital status, PIR, physical activity, smoking, total energy intake, eGFR, drinking, BMI, DM, CVD, cancer, anemia treatment, gout, COPD, HB, dialysis, CRP, and hypothyroidism for all‐cause mortality.

Adjusted for age, race, education level, marital status, PIR, physical activity, smoking, eGFR, drinking, DM, cancer, anemia treatment, gout, COPD, HB, dialysis, CRP, and hypothyroidism for CVD‐specific mortality.

Higher adherence to AHEI‐2010 was related to lower risks of both all‐cause mortality (HR = 0.74, 95% CI: [0.58–0.94]) and CVD‐specific mortality (HR = 0.73 95% CI: [0.54–0.99]) in patients who were normal or overweight. Besides, higher MED score was only linked to a decreased risk of all‐cause mortality (HR = 0.74, 95% CI: [0.62–0.88]).

In HTN patients with CVD, high adherence to AHEI‐2010 was associated with a decreased risk of CVD‐specific mortality (HR = 0.59, 95% CI: [0.40–0.87]). Differently, patients without CVD who had high HEI‐2015 (HR = 0.81, 95% CI: [0.67–0.98]) and AHEI‐2010 (HR = 0.77, 95% CI: [0.63–0.94]) were seemed to have a decreased risk of all‐cause mortality.

AHEI‐2010 was also found to be linked to decreased risks of all‐cause mortality and CVD‐specific mortality in patients without DM and with dyslipidemia (all *p* < .05). Moreover, higher MED score was only related to lower risk of all‐cause mortality in patients with dyslipidemia (HR = 0.82, 95% CI: [0.67–0.99]).

## 4 DISCUSSION

The current study explored the association between different dietary patterns and all‐cause mortality and CVD‐specific mortality in patients with HTN. Our results showed that high AHEI‐2010 and MED scores were linked to decreased risks of both all‐cause mortality and CVD‐specific mortality. Also, these relationships in patients with different age, BMI, CVD, DM, and dyslipidemia were different.

Although there are few reports on the relationship between different diet quality scores and the prognosis in patients with HTN, previous research can still be instructive. Zhong et al.^20^ analyzed data from 6 prospective cohorts collected in 1985–2016 to assess the role of diet quality in the long‐term absolute risks for CVD and mortality in adults. The results showed that participants who consumed a diet with the highest quintile of AHEI‐2010 score or DASH score had lower long‐term absolute risks for incident of CVD and all‐cause mortality.^20^ In a large‐sample prospective study in the Middle East, researchers found that individuals with the highest quintile of dietary scores including AHEI‐2010, MED, and DASH, compared with that of the lowest quintile dietary scores, had a significantly decreased all‐cause mortality, and a reduced CVD‐specific mortality was found for those who had high AHEI‐2010 and DASH scores.^21^ Another prospective cohort study of participants from the Atherosclerosis Risk in Communities Study showed that compared with participants in the lowest quintile of dietary scores including HEI‐2015, AHEI‐2010, MED, and DASH, participants in the highest quintile had lower risks of incident CVD, CVD‐specific mortality, and all‐cause mortality.^10^ In the current study, we found patients with HTN in the highest tertile of AHEI‐2010 and MED score had lower risks of both all‐cause mortality and CVD‐specific mortality. Differently, we could not find significant association between other dietary scores (including HEI‐2015 and DASH) and all‐cause mortality or CVD‐specific mortality in this study. The possible reasons may be the study population was different from the previous study, or patients with HTN usually received appropriate treatment, or they focused more on keeping a hypotensive lifestyle because there are many factors that affect the BP.

Greater adherence to HEI‐2015 has been reported to be inversely correlated with the risk of incident HTN, and among its components, a higher score of total fruits, fatty acids, and Na was associated with a reduced risk of incident HTN.^22^ The AHEI, modeled on the original HEI, was constructed according to extensive epidemiological evidence for noncommunicable diseases prevention.^23^ Although HEI and AHEI diet scores were developed for slightly different purposes, they both emphasize on high intake of fruits, vegetables, monounsaturated fat, legumes, whole grains, and low consumption of saturated fatty acids and recommends lowers intakes for added sugars, refined grains, and Na, which associated with the increasing risk of HTN.^24^, ^25^ Diets rich in fiber, low energy density, and low glycemic load are protective against HTN.^26^ The favorable impacts of vegetables/fruits on HTN could be attributed to potential healthy constituents such as phytochemicals,^27^ vitamins,^28^ Mg, and K^29^ and antioxidants,^30^ which have been independently related to a reduction in BP. The consumption of nuts and soy and moderate alcohol intake appeared to be the most important independent contributors to decreased mortality risk.^31^ The relatively high consumption of nuts, olive oil and moderate intake of red wine during meals (which can be considered a primarily plant‐based diet), makes the MED diet unique and different from the other healthy diet patterns.^32^ Moreover, it has also been reported that BP can be decreased when individuals had high adherence to MED diet.^33^, ^34^ These may explain our finding that high AHEI‐2010 and MED scores were associated with low risks of all‐cause mortality and CVD‐specific mortality.

Due to HTN may be a result of and can be influenced by multiple factors, we further performed subgroup analyses. Age, low educational attainment, overweight, obesity, abdominal obesity, and high cholesterol and blood glucose were strongly associated with HTN.^35^ In age subgroups, the highest tertiles of HEI‐2015, AHEI‐2010, and MED score were all associated with both all‐cause mortality and CVD‐specific mortality in HTN patients who aged ≥65 years old. HTN is particularly frequent in old age. The average age of our study population was 53.45 years old, and there was a significant difference between survival and mortality groups which the difference was over 10 years. The potential mechanisms of the roles of these dietary patterns in elderly persons with HTN to be more significant compared with that in youngers are needed further exploration. HTN patients who were normal or overweight other than obese had high AHEI‐2010 were seemed to have decreased risks of all‐cause mortality and CVD‐specific mortality. Our study was in line with previous studies. Mohebbi et al.^36^ considered BMI (normal and overweight) can be a predictor of adherence to diet. High sugar and/or refined grains foods can alter HTN risk through mechanisms related to obesity, metabolic dysregulation, vascular dysfunction, and salt retention.^37^ However, we did not find any difference between genders in the relationships of different dietary patterns and mortality. The potential explanations for the gender difference have been reported, which could be the higher carbohydrate intakes typically observed among women, and evidence that females have increased sensitivity to insulin in skeletal muscle than men, leading to higher levels of intramuscular fat and subsequently increased HTN risk.^38^ Moreover, we also observed that different dietary patterns were linked to the mortality in HTN patients with different complications including CVD, DM, and dyslipidemia. High BP not only results in deleterious mechanical stress on blood vessels but also on the myocardium, leading to the development of hypertensive heart disease and congestive heart failure.^39^ The potential pathways of action may be dietary fiber can reduce LDL cholesterol and TG uptake to improve the elasticity of blood vessel walls to decrease vascular resistance and maintain adequate tissue perfusion.^40^ High‐fiber foods such as vegetables also contain beneficial nutrients metabolized into compounds which may improve BP through greater bioavailability for use in vasodilation.^41^ Furthermore, recent studies have found some supports for reductions in body weight with higher fiber intakes, as shown in evidence synthesis of the general population and those with DM, with weight loss beneficial in the treatment and prevention of HTN.^42^, ^43^ Herein, we considered that our study may partly provide some new ideas for the public policies related to dietary recommendation to improve health in patients with HTN, because the traditional DASH dietary may not consider the difference among all interindividual health conditions. Recommendation of different dietary patterns to different populations or providing a combined dietary pattern may be more suitable for high‐risk patients with HTN.

### 4.1 Strengths and limitations

The present study used samples from the NHANES database which is representative. In this cohort study, the follow‐up time was long, and the mortality information was obtained from the death certificate so that the outcome assessment was accurate. Besides, we assessed different dietary scores which related to HTN may provide dietary guidance for prognostic management of patients with HTN. However, we only dietary intake data were collected at baseline, which may not reflect long‐term dietary patterns and changes during the follow‐up, so we chose the 2‐day average of dietary survey data. Although confounders such as socioeconomic factors and chronic diseases were adjusted, other potential confounders such as disease status and treatment during follow‐up were difficult to adjust.

### 4.2 Future direction

Further prospective large‐sample cohort studies are needed to explore the association between different dietary patterns and prognoses in patients with HTN and the potential mechanisms, which may provide some guidelines on dietary patterns for the management and treatment of high‐risk populations.

## 5 CONCLUSION

High AHEI‐2010 and MED scores were related to decreased risks of both all‐cause mortality and CVD‐specific mortality in patients with HTN.

## CONFLICT OF INTEREST STATEMENT

The authors declare no conflict of interest.

## Supporting information

Supporting information.

Supporting information.

## Data Availability

The data sets used and/or analyzed during the current study are available from the NHANES database, https://wwwn.cdc.gov/nchs/nhanes/.
