# Supplementary material for: Effects of dietary patterns on the all‐cause mortality and cardiovascular disease mortality in patients with hypertension: A cohort study based on the NHANES database
Source: Clin Cardiol. 2023 Aug 16;46(11):1353–70. doi: 10.1002/clc.24118 (PMC10642326; doi:10.1002/clc.24118)
Supplement: Supplementary file 1 — Supporting information. [file CLC-46-1353-s001.docx]

Table S1. Characteristics of HTN patients before and after interpolation of missing data

| Variables | After interpolation  (n=21759) | Before interpolation  (n=21759) | Statistics | *P* |
| --- | --- | --- | --- | --- |
| Education level, n (%) |  |  | χ^2^=0.002 | 1.000 |
| Less Than 9th Grade | 2414 (11.10) | 2415 (11.10) |  |  |
| 9-11th Grade | 3189 (14.67) | 3194 (14.68) |  |  |
| High School Grad/GED or Equivalent | 5342 (24.57) | 5344 (24.56) |  |  |
| Some College or AA degree | 6463 (29.72) | 6469 (29.73) |  |  |
| College Graduate or above | 4335 (19.94) | 4337 (19.93) |  |  |
| Marital status, n (%) |  |  | χ^2^=0.002 | 1.000 |
| Married | 11682 (53.72) | 11690 (53.72) |  |  |
| Widowed | 2074 (9.54) | 2074 (9.54 |  |  |
| Divorced | 2807 (12.91) | 2807 (12.91) |  |  |
| Separated | 824 (3.79) | 824 (3.79) |  |  |
| Never married | 2895 (13.31) | 2898 (13.32) |  |  |
| Living with partner | 1466 (6.74) | 1466 (6.74) |  |  |
| Waistline, cm, Mean±SD | 103.44 ± 16.30 | 103.63 ± 16.57 | t=-1.21 | 0.227 |
| BMI, kg/m^2^, Mean±SD | 30.69 ± 7.48 | 30.70 ± 7.49 | t=-0.12 | 0.905 |
| Cr, mg, M (Q_1_, Q_3_) | 0.90 (0.77, 1.08) | 0.90 (0.77, 1.08) | Z=0.108 | 0.914 |
| HB, g/dL, Mean±SD | 14.19 ± 1.62 | 14.19 ± 1.62 | t=0.40 | 0.691 |
| Cancer, n (%) |  |  | χ^2^=0.001 | 0.981 |
| No | 19357 (89.05) | 19375 (89.04) |  |  |
| Yes | 2380 (10.95) | 2384 (10.96) |  |  |
| Asthma, n (%) |  |  | χ^2^=0.001 | 0.980 |
| No | 18485 (85.04) | 18501 (85.03) |  |  |
| Yes | 3253 (14.96) | 3258 (14.97) |  |  |

HTN: hypertension, SD: standard deviation, M: medium, Q_1_:1st quartile, Q_3_:3st quartile, BMI: body mass index, Cr: creatinine, HB: hemoglobin.

t: t test, χ^2^: chi-square test, Z: rank sum test

Table S2. Covariates related to all-cause mortality and CVD-specific mortality

| Variables | All-cause mortality | | CVD-specific mortality | |
| --- | --- | --- | --- | --- |
|  | HR (95% CI) | *P* | HR (95% CI) | *P* |
| Age | 1.09 (1.08-1.10) | <0.001 | 1.11 (1.10-1.13) | <0.001 |
| Gender |  |  |  |  |
| Male | Ref |  | Ref |  |
| Female | 1.03 (0.94-1.14) | 0.501 | 1.04 (0.83-1.31) | 0.711 |
| Race |  |  |  |  |
| Mexican American | Ref |  | Ref |  |
| Other Hispanic | 1.00 (0.67-1.49) | 0.992 | 1.80 (0.83-3.89) | 0.135 |
| Non-Hispanic White | 2.15 (1.71-2.70) | <0.001 | 2.20 (1.30-3.70) | 0.003 |
| Non-Hispanic Black | 2.00 (1.60-2.49) | <0.001 | 2.65 (1.56-4.50) | <0.001 |
| Other Race - including multi-racial | 1.23 (0.80-1.89) | 0.343 | 1.06 (0.53-2.10) | 0.871 |
| Education level |  |  |  |  |
| Less Than 9th Grade | Ref |  | Ref |  |
| 9-11th Grade | 0.74 (0.58-0.96) | 0.022 | 0.73 (0.55-0.99) | 0.043 |
| High School Grad/GED or Equivalent | 0.54 (0.44-0.68) | <0.001 | 0.59 (0.43-0.80) | <0.001 |
| Some College or AA degree | 0.41 (0.33-0.51) | <0.001 | 0.36 (0.27-0.50) | <0.001 |
| College Graduate or above | 0.28 (0.20-0.39) | <0.001 | 0.28 (0.18-0.43) | <0.001 |
| Marital status |  |  |  |  |
| Married | Ref |  | Ref |  |
| Widowed | 5.99 (5.27-6.80) | <0.001 | 7.82 (6.35-9.64) | <0.001 |
| Divorced | 1.52 (1.16-2.00) | 0.002 | 1.32 (0.82-2.13) | 0.260 |
| Separated | 1.23 (0.80-1.88) | 0.351 | 1.79 (0.76-4.22) | 0.181 |
| Never married | 0.85 (0.64-1.14) | 0.279 | 0.88 (0.58-1.34) | 0.555 |
| Living with partner | 1.05 (0.68-1.64) | 0.820 | 0.78 (0.31-1.96) | 0.596 |
| PIR |  |  |  |  |
| ≤1 | Ref |  | Ref |  |
| >1 | 0.62 (0.50-0.78) | <0.001 | 0.65 (0.51-0.83) | <0.001 |
| Physical activity | 0.99 (0.99-0.99) | <0.001 | 0.99 (0.99-0.99) | <0.001 |
| Total energy intake | 0.99 (0.99-0.99) | <0.001 | 0.99 (0.99-0.99) | <0.001 |
| Smoking |  |  |  |  |
| Yes | Ref |  | Ref |  |
| No | 0.57 (0.49-0.66) | <0.001 | 0.77 (0.65-0.91) | 0.003 |
| eGFR | 0.96 (0.96-0.97) | <0.001 | 0.96 (0.95-0.96) | <0.001 |
| Drinking |  |  |  |  |
| Yes | Ref |  | Ref |  |
| No | 1.40 (1.22-1.60) | <0.001 | 1.73 (1.37-2.19) | <0.001 |
| BMI | 0.98 (0.97-0.99) | 0.002 | 0.99 (0.97-1.01) | 0.294 |
| DM |  |  |  |  |
| No | Ref |  | Ref |  |
| Yes | 2.59 (2.23-3.02) | <0.001 | 2.64 (2.12-3.28) | <0.001 |
| CVD |  |  |  |  |
| No | Ref |  | Ref |  |
| Yes | 4.85 (4.11-5.73) | <0.001 | 7.16 (5.54-9.27) | <0.001 |
| Dyslipidemia |  |  |  |  |
| No | Ref |  | Ref |  |
| Yes | 1.26 (1.06-1.50) | 0.008 | 1.33 (0.98-1.81) | 0.070 |
| Cancer |  |  |  |  |
| No | Ref |  | Ref |  |
| Yes | 2.63 (2.28-3.03) | <0.001 | 2.44 (1.93-3.07) | <0.001 |
| Asthma |  |  |  |  |
| No | Ref |  | Ref |  |
| Yes | 0.99 (0.72-1.37) | 0.965 | 0.88 (0.68-1.12) | 0.296 |
| Anemia treatment |  |  |  |  |
| No | Ref |  | Ref |  |
| Yes | 2.95 (2.36-3.69) | <0.001 | 4.06 (2.99-5.52) | <0.001 |
| Gout |  |  |  |  |
| No | Ref |  | Ref |  |
| Yes | 2.14 (1.81-2.55) | <0.001 | 2.47 (1.95-3.14) | <0.001 |
| Unknown | 0.46 (0.35-0.60) | <0.001 | 0.40 (0.27-0.61) | <0.001 |
| COPD |  |  |  |  |
| No | Ref |  | Ref |  |
| Yes | 4.46 (3.35-5.95) | <0.001 | 3.75 (1.84-7.67) | <0.001 |
| Unknown | 0.71 (0.57-0.88) | 0.002 | 0.74 (0.51-1.06) | 0.102 |
| Platelet | 1.00 (0.92-1.09) | 0.964 | 0.97 (0.86-1.09) | 0.603 |
| HB | 0.81 (0.78-0.85) | <0.001 | 0.75 (0.70-0.80) | <0.001 |
| Dialysis |  |  |  |  |
| No | Ref |  | Ref |  |
| Yes | 3.27 (1.90-5.64) | <0.001 | 2.83 (1.28-6.24) | 0.010 |
| Unknown | 0.34 (0.25-0.46) | <0.001 | 0.31 (0.20-0.50) | <0.001 |
| CRP |  |  |  |  |
| No | Ref |  | Ref |  |
| Yes | 1.64 (1.29-2.08) | <0.001 | 1.21 (0.91-1.61) | 0.199 |
| Unknown | 2.22 (1.75-2.82) | <0.001 | 2.11 (1.53-2.93) | <0.001 |
| Hypothyroidism |  |  |  |  |
| No | Ref |  | Ref |  |
| Yes | 1.46 (0.98-2.17) | 0.062 | 1.34 (0.81-2.21) | 0.254 |
| Unknown | 0.65 (0.54-0.78) | <0.001 | 0.57 (0.43-0.76) | <0.001 |

CVD: cardiovascular disease, HR: hazard ratio, CI: confidence interval, Ref: reference, PIR: poverty-to-income ratio, eGFR: estimated glomerular filtration rate, BMI: body mass index, DM: diabetes mellitus, COPD: chronic obstructive pulmonary disease, HB: hemoglobin, CRP: C-reactive protein.
