# Supplementary material for: Effects of dietary patterns on the all‐cause mortality and cardiovascular disease mortality in patients with hypertension: A cohort study based on the NHANES database
Source: Clin Cardiol. 2023 Aug 16;46(11):1353–70. doi: 10.1002/clc.24118 (PMC10642326; doi:10.1002/clc.24118)
Supplement: Supplementary file 2 — Supporting information. [file CLC-46-1353-s002.docx]

library(survey)

library(dplyr)

dir <- dirname(rstudioapi::getActiveDocumentContext()$path)

setwd(dir)

##1.3 function

outrtf <- function(data,cov,path){

c <- flextable(

data=data,

cwidth = 2,

cheight = 0.1,

defaults = list(),

theme_fun = theme_booktabs

)

library(xtable)

library(flextable)

library(officer)

c <- footnote( c, i = 1, j = 1:3,

value = as_paragraph(

c("OR：Odds Ratio",

"CI: Confidence Interval",

paste0("Adjusted",cov))),

ref_symbols = c("", "", ""),

part = "header")

c <- italic(c,j=3,part="header")

c <- fontsize(c, i = NULL, j = NULL, size = 11, part = "body")

c <- font(c,fontname="SimSun",part="all")

c <- font(c,fontname="Times New Roman",part="all")

c <- hline_top(c,border=fp_border(color="black",width=1.5),part="header")

save_as_docx(c,path=path)

}

##1.4 read data

cleaned <- as.data.frame(haven::read_sas("analysis_data.sas7bdat"))

cleaned_v2 <- as.data.frame(haven::read_sas("analysis_data_final.sas7bdat"))

####—————————————————————————————————————————————————————2. data cleaning—————————————————————————————————————————————————————####

#ahei_2010 计算

cleaned$ahei_1 <- cut(cleaned$tot_vege,

breaks=(c(-100,5/10,5*2/10,5*3/10,5*4/10,5*5/10,5*6/10,5*7/10,5*8/10,5*9/10,5,100000)),

labels=c(0,1,2,3,4,5,6,7,8,9,10),right=F)

cleaned$ahei_2 <- cut(cleaned$tot_fruit,

breaks=(c(-100,4/10,4*2/10,4*3/10,4*4/10,4*5/10,4*6/10,4*7/10,4*8/10,4*9/10,4,100000)),

labels=c(0,1,2,3,4,5,6,7,8,9,10),right=F)

cleaned$ahei_3 <- ifelse(cleaned$gender==1,

cut(cleaned$whole_grain,

breaks=(c(-100,5/10,5*2/10,5*3/10,5*4/10,5*5/10,5*6/10,5*7/10,5*8/10,5*9/10,5,100000)),

labels=c(0,1,2,3,4,5,6,7,8,9,10),right=F),

cut(cleaned$whole_grain,

breaks=(c(-100,6/10,6*2/10,6*3/10,6*4/10,6*5/10,6*6/10,6*7/10,6*8/10,6*9/10,6,100000)),

labels=c(0,1,2,3,4,5,6,7,8,9,10),right=F))-1

cleaned$ahei_4 <- cut(cleaned$fruit_juice,

breaks=(c(-100,1/10,1*2/10,1*3/10,1*4/10,1*5/10,1*6/10,1*7/10,1*8/10,1*9/10,1,100000)),

labels=c(10,9,8,7,6,5,4,3,2,1,0))

cleaned$ahei_5 <- cut(cleaned$nut,

breaks=(c(-100,1/10,1*2/10,1*3/10,1*4/10,1*5/10,1*6/10,1*7/10,1*8/10,1*9/10,1,100000)),

labels=c(0,1,2,3,4,5,6,7,8,9,10),right=F)

cleaned$ahei_6 <- cut(cleaned$meat,

breaks=(c(-100,1.5/10,1.5*2/10,1.5*3/10,1.5*4/10,1.5*5/10,1.5*6/10,1.5*7/10,1.5*8/10,1.5*9/10,1.5,100000)),

labels=c(10,9,8,7,6,5,4,3,2,1,0))

cleaned$ahei_7 <- cut(cleaned$n3_,

breaks=(c(0,2.5/10,2.5*2/10,2.5*3/10,2.5*4/10,2.5*5/10,2.5*6/10,2.5*7/10,2.5*8/10,2.5*9/10,2.5,100000)),

labels=c(0,1,2,3,4,5,6,7,8,9,10))

cleaned$ahei_8 <- cut(cleaned$sodium,

breaks=(c(-100,quantile(cleaned$sodium,0.1),quantile(cleaned$sodium,0.2),

quantile(cleaned$sodium,0.3),quantile(cleaned$sodium,0.4),quantile(cleaned$sodium,0.5),

quantile(cleaned$sodium,0.6),quantile(cleaned$sodium,0.7),quantile(cleaned$sodium,0.8),

quantile(cleaned$sodium,0.9),max(cleaned$sodium))),

labels=c(9,8,7,6,5,4,3,2,1,0))

cleaned$ahei_9 <- ifelse(cleaned$gender==1,

cut(cleaned$alchol/14,

breaks=(c(0,0.5*1/10,0.5*2/10,0.5*3/10,0.5*4/10,0.5*5/10,0.5*6/10,0.5*7/10,0.5*8/10,0.5*9/10,0.5,2,(1.5*1/10)+2,(1.5*2/10)+2,(1.5*3/10)+2,(1.5*4/10)+2,(1.5*5/10)+2,(1.5*6/10)+2,(1.5*7/10)+2,(1.5*8/10)+2,(1.5*9/10)+2,3.5,100000)),

labels=c(0,1,2,3,4,5,6,7,8,9,10,9,8,7,6,5,4,3,2,1,0,0),right=F),

cut(cleaned$alchol/14,

breaks=(c(0,0.5*1/10,0.5*2/10,0.5*3/10,0.5*4/10,0.5*5/10,0.5*6/10,0.5*7/10,0.5*8/10,0.5*9/10,0.5,1.5,(1*1/10)+1.5,(1*2/10)+1.5,(1*3/10)+1.5,(1*4/10)+1.5,(1*5/10)+1.5,(1*6/10)+1.5,(1*7/10)+1.5,(1*8/10)+1.5,(1*9/10)+1.5,2.5,100000)),

labels=c(0,1,2,3,4,5,6,7,8,9,10,9,8,7,6,5,4,3,2,1,0,0),right=F))

cleaned$ahei_9 <- ifelse(cleaned$alchol==0,2.5,cleaned$ahei_9)

cleaned$ahei_10 <- cut((cleaned$total_polyunsaturated*9/cleaned$energy)*100,

breaks=(c(0,2,(8/9)+2,(8*2/9)+2,(8*3/9)+2,(8*4/9)+2,(8*5/9)+2,(8*6/9)+2,(8*7/9)+2,(8*8/9)+2,10,100000)),

labels=c(0,1,2,3,4,5,6,7,8,9,10))

writexl::write_xlsx(cleaned,"analysis_data_v2.xlsx")

cleaned_v2$race <- cleaned_v2$race %>% factor

cleaned_v2$gender <- cleaned_v2$gender %>% factor

cleaned_v2$education <- cleaned_v2$education %>% factor

cleaned_v2$education_r <- cleaned_v2$education_r %>% factor

cleaned_v2$marriage <- cleaned_v2$marriage %>% factor

cleaned_v2$smoke <- cleaned_v2$smoke %>% factor

cleaned_v2$drink <- cleaned_v2$drink %>% factor

cleaned_v2$cvd_status <- cleaned_v2$cvd_status %>% factor

cleaned_v2$diabete_status <- cleaned_v2$diabete_status %>% factor

cleaned_v2$high_lipid <- cleaned_v2$high_lipid %>% factor

cleaned_v2$age_r <- factor(ifelse(cleaned_v2$age>65,3,

ifelse(cleaned_v2$age>40&cleaned_v2$age<=65,2,1)))

cleaned_v2$cancer <- cleaned_v2$cancer %>% factor

cleaned_v2$asthma <- cleaned_v2$asthma %>% factor

cleaned_v2$Anemia_trt <- cleaned_v2$Anemia_trt %>% factor

cleaned_v2$gout <- cleaned_v2$gout %>% factor

cleaned_v2$copd <- cleaned_v2$copd %>% factor

cleaned_v2$jiajian <- cleaned_v2$jiajian %>% factor

cleaned_v2$hiv_test <- cleaned_v2$hiv_test %>% factor

cleaned_v2$drug_abuse <- cleaned_v2$drug_abuse %>% factor

cleaned_v2$crp_r <- cleaned_v2$crp_r %>% factor

cleaned_v2$dialysis <- cleaned_v2$dialysis %>% factor

cleaned_v2$pir_r <- ifelse(cleaned_v2$pir<=1,1,2) %>% as.factor

cleaned_v2$hei_2015_r <- cut(cleaned_v2$TOTAL_SCORE,

breaks = c(0,

quantile(cleaned_v2$TOTAL_SCORE,0.33),

quantile(cleaned_v2$TOTAL_SCORE,0.66),

999),right=T,

labels=c(1,2,3)) %>% as.factor

cleaned_v2$ahei_2010_r <- cut(cleaned_v2$ahei_2010,

breaks = c(0,

quantile(cleaned_v2$ahei_2010,0.33),

quantile(cleaned_v2$ahei_2010,0.66),

999),right=T,

labels=c(1,2,3)) %>% as.factor

cleaned_v2$dash_r <- cut(cleaned_v2$dash_score,

breaks = c(0,

quantile(cleaned_v2$dash_score,0.33),

quantile(cleaned_v2$dash_score,0.66),

999),right=T,

labels=c(1,2,3)) %>% as.factor

cleaned_v2$Med_score_r <- cut(cleaned_v2$Med_score,

breaks = c(0,

quantile(cleaned_v2$Med_score,0.33),

quantile(cleaned_v2$Med_score,0.66),

999),right=F,

labels=c(1,2,3)) %>% as.factor

####—————————————————————————————————————————————————————3. data analysis—————————————————————————————————————————————————————####

library(survminer)

Surv_sft1 <- survfit(Surv(cleaned_v2$time,cleaned_v2$status)~factor(cleaned_v2$hei_2015_r))

Surv_sft2 <- survfit(Surv(cleaned_v2$time,cleaned_v2$status)~factor(cleaned_v2$ahei_2010_r))

Surv_sft3 <- survfit(Surv(cleaned_v2$time,cleaned_v2$status)~factor(cleaned_v2$dash_r))

Surv_sft4 <- survfit(Surv(cleaned_v2$time,cleaned_v2$status)~factor(cleaned_v2$Med_score_r))

Surv_sft1_cvd <- survfit(Surv(cleaned_v2$time,cleaned_v2$status_cvd)~factor(cleaned_v2$hei_2015_r))

Surv_sft2_cvd <- survfit(Surv(cleaned_v2$time,cleaned_v2$status_cvd)~factor(cleaned_v2$ahei_2010_r))

Surv_sft3_cvd <- survfit(Surv(cleaned_v2$time,cleaned_v2$status_cvd)~factor(cleaned_v2$dash_r))

Surv_sft4_cvd <- survfit(Surv(cleaned_v2$time,cleaned_v2$status_cvd)~factor(cleaned_v2$Med_score_r))

pdf("KM_hei_all.pdf",width = 8,height=6)

ggsurvplot(Surv_sft1, pval = TRUE,

conf.int = F,censor=F, xlab='Follow Up time (Days)',

surv.median.line = "none",

ggtheme = theme_bw() ,

font.families="serif",ylim=c(0.5,1),legend="right",

palette = "npg", legend.title='HEI-2015',

legend.labs=c('<=46.64','(46.64, 58.28]',' >58.28'),data = cleaned_v2)

dev.off()

pdf("KM_ahei_all.pdf",width = 8,height=6)

ggsurvplot(Surv_sft2, pval = TRUE,

conf.int = F,censor=F, xlab='Follow Up time (Days)',

surv.median.line = "none",

ggtheme = theme_bw() ,

font.families="serif",ylim=c(0.5,1),

palette = "npg", legend.title='AHEI 2010',legend="right",

legend.labs=c('<=30.5','(30.5, 39]',' >39'),data = cleaned_v2)

dev.off()

pdf("KM_dash_all.pdf",width = 8,height=6)

ggsurvplot(Surv_sft3, pval = TRUE,

conf.int = F,censor=F, xlab='Follow Up time (Days)',

surv.median.line = "none",

ggtheme = theme_bw() ,

font.families="serif",ylim=c(0.5,1),legend="right",

palette = "npg", legend.title='Dash score',

legend.labs=c('<=1.5','(1.5, 2.5]',' >2.5'),data = cleaned_v2)

dev.off()

pdf("KM_med_all.pdf",width = 8,height=6)

ggsurvplot(Surv_sft4, pval = TRUE,

conf.int = F,censor=F, xlab='Follow Up time (Days)',

surv.median.line = "none",

ggtheme = theme_bw() ,

font.families="serif",ylim=c(0.5,1),legend="right",

palette = "npg", legend.title='Med score ',

legend.labs=c('<=3','(3,5]',' >5'),data = cleaned_v2)

dev.off()

pdf("KM_hei_cvd.pdf",width = 8,height=6)

ggsurvplot(Surv_sft1_cvd, pval = TRUE,

conf.int = F,censor=F, xlab='Follow Up time (Days)',

surv.median.line = "none",

ggtheme = theme_bw() ,

font.families="serif",ylim=c(0.5,1),legend="right",

palette = "npg", legend.title='HEI-2015',

legend.labs=c('<=46.64','(46.64, 58.28]',' >58.28'),data = cleaned_v2)

dev.off()

pdf("KM_ahei_cvd.pdf",width = 8,height=6)

ggsurvplot(Surv_sft2_cvd, pval = TRUE,

conf.int = F,censor=F, xlab='Follow Up time (Days)',

surv.median.line = "none",

ggtheme = theme_bw() ,

font.families="serif",ylim=c(0.5,1),legend="right",

palette = "npg", legend.title='AHEI 2010',

legend.labs=c('<=30.5','(30.5, 39]',' >39'),data = cleaned_v2)

dev.off()

pdf("KM_Dash_cvd.pdf",width = 8,height=6)

ggsurvplot(Surv_sft3_cvd, pval = TRUE,

conf.int = F,censor=F, xlab='Follow Up time (Days)',

surv.median.line = "none",

ggtheme = theme_bw() ,legend="right",

font.families="serif",ylim=c(0.5,1),

palette = "npg", legend.title='Dash score',

legend.labs=c('<=1.5','(1.5, 2.5]',' >2.5'),data = cleaned_v2)

dev.off()

pdf("KM_med_cvd.pdf",width = 8,height=6)

ggsurvplot(Surv_sft4_cvd, pval = TRUE,

conf.int = F,censor=F, xlab='Follow Up time (Days)',

surv.median.line = "none",

ggtheme = theme_bw() ,legend="right",

font.families="serif",ylim=c(0.5,1),

palette = "npg", legend.title='Med score ',

legend.labs=c('<=3','(3,5]',' >5'),data = cleaned_v2)

dev.off()

####---------------------------------------all cause----------------------------------------####

###screening

source("get_cox_weight.R")

screen1 <- get_cox_weight(data=cleaned_v2,

var='age gender race education marriage pir_r met smoke energy egfr drink bmi wc diabete_status cvd_status high_lipid cancer asthma Anemia_trt gout copd platelet Hemoglobin dialysis crp_r jiajian',

covariate='',

response='status',

follow_up_time= 'time',

out="No") %>% as.data.frame()

cov <- paste(screen1 %>% filter(p2==1) %>% select(first_col)%>% as.vector() %>% unlist ,collapse=" ")

screen2 <- get_cox_weight(data=cleaned_v2,

var='age gender race education marriage pir_r met smoke energy egfr drink bmi wc diabete_status cvd_status high_lipid cancer asthma Anemia_trt gout copd platelet Hemoglobin dialysis crp_r jiajian',

covariate='',

response='status_cvd',

follow_up_time= 'time',

out="No") %>% as.data.frame()

cov_cvd <- paste(screen2 %>% filter(p2==1) %>% select(first_col)%>% as.vector() %>% unlist ,collapse=" ")

res1 <- get_cox_weight(data=cleaned_v2,

var='hei_2015_r ahei_2010_r dash_r Med_score_r',

covariate='',

response='status',

follow_up_time= 'time',

out="No")

res2 <- get_cox_weight(data=cleaned_v2,

var='hei_2015_r ahei_2010_r dash_r Med_score_r',

covariate='age race education marriage',

response='status',

follow_up_time= 'time',

out="No")

res3 <- get_cox_weight(data=cleaned_v2,

var='hei_2015_r ahei_2010_r dash_r Med_score_r',

covariate=cov,

response='status',

follow_up_time= 'time',

out="No")

res_sum <- cbind(res1[,c(1,2,4)],res2[,c(2,4)],res3[,c(2,4)])

##subgroup

cov_age <- "age race education marriage pir_r met smoke egfr drink bmi diabete_status cvd_status high_lipid cancer Anemia_trt gout copd Hemoglobin dialysis crp_r jiajian"

cov_gender <- "age race education marriage pir_r met energy smoke egfr drink bmi diabete_status cvd_status high_lipid cancer Anemia_trt gout copd Hemoglobin dialysis crp_r jiajian"

cov_diabetes <- "age race education marriage pir_r met smoke egfr drink bmi cvd_status high_lipid cancer Anemia_trt gout copd Hemoglobin dialysis crp_r jiajian"

cov_cvd<- "age race education marriage pir_r met smoke egfr drink bmi diabete_status high_lipid cancer Anemia_trt gout copd Hemoglobin dialysis crp_r jiajian"

cov_hlipid <- "age race education marriage pir_r met smoke egfr drink bmi diabete_status cvd_status cancer Anemia_trt gout copd Hemoglobin dialysis crp_r jiajian"

cov_bmi <-"age race education marriage pir_r met smoke egfr drink diabete_status cvd_status high_lipid cancer Anemia_trt gout copd Hemoglobin dialysis crp_r jiajian"

cleaned_v2_age1 <- cleaned_v2 %>% filter(age<65)

cleaned_v2_age2 <- cleaned_v2 %>% filter(age>=65)

cleaned_v2_gender1 <- cleaned_v2 %>% filter(gender==1)

cleaned_v2_gender2 <- cleaned_v2 %>% filter(gender==2)

cleaned_v2_diabetes1<- cleaned_v2 %>% filter(diabete_status==1)

cleaned_v2_diabetes2 <- cleaned_v2 %>% filter(diabete_status==0)

cleaned_v2_cvd1 <- cleaned_v2 %>% filter(CVD==1)

cleaned_v2_cvd2 <- cleaned_v2 %>% filter(CVD==0)

cleaned_v2_high_lipid1 <- cleaned_v2 %>% filter(high_lipid==1)

cleaned_v2_high_lipid2 <- cleaned_v2 %>% filter(high_lipid==0)

cleaned_v2_BMI1 <- cleaned_v2 %>% filter(bmi<30)

cleaned_v2_BMI2 <- cleaned_v2 %>% filter(bmi>=30)

bmi1 <- get_cox_weight(data=cleaned_v2_BMI1,

var='hei_2015_r ahei_2010_r dash_r Med_score_r',

covariate=cov_bmi,

response='status',

follow_up_time= 'time',

out="No")

bmi2 <- get_cox_weight(data=cleaned_v2_BMI2,

var='hei_2015_r ahei_2010_r dash_r Med_score_r',

covariate=cov_bmi,

response='status',

follow_up_time= 'time',

out="No")

age1 <- get_cox_weight(data=cleaned_v2_age1,

var='hei_2015_r ahei_2010_r dash_r Med_score_r',

covariate=cov_age,

response='status',

follow_up_time= 'time',

out="No")

age2 <- get_cox_weight(data=cleaned_v2_age2,

var='hei_2015_r ahei_2010_r dash_r Med_score_r',

covariate=cov_age,

response='status',

follow_up_time= 'time',

out="No")

gender1 <- get_cox_weight(data=cleaned_v2_gender1,

var='hei_2015_r ahei_2010_r dash_r Med_score_r',

covariate=cov_gender,

response='status',

follow_up_time= 'time',

out="No")

gender2 <- get_cox_weight(data=cleaned_v2_gender2,

var='hei_2015_r ahei_2010_r dash_r Med_score_r',

covariate="age race education marriage pir_r met energy smoke egfr drink bmi diabete_status cvd_status high_lipid cancer Anemia_trt gout copd dialysis crp_r jiajian",

response='status',

follow_up_time= 'time',

out="No")

dabetes1<- get_cox_weight(data=cleaned_v2_diabetes1,

var='hei_2015_r ahei_2010_r dash_r Med_score_r',

covariate=cov_diabetes,

response='status',

follow_up_time= 'time',

out="No")

dabetes2 <- get_cox_weight(data=cleaned_v2_diabetes2,

var='hei_2015_r ahei_2010_r dash_r Med_score_r',

covariate=cov_diabetes,

response='status',

follow_up_time= 'time',

out="No")

cvd1<- get_cox_weight(data=cleaned_v2_cvd1,

var='hei_2015_r ahei_2010_r dash_r Med_score_r',

covariate=cov_cvd,

response='status',

follow_up_time= 'time',

out="No")

cvd2<- get_cox_weight(data=cleaned_v2_cvd2,

var='hei_2015_r ahei_2010_r dash_r Med_score_r',

covariate=cov_cvd,

response='status',

follow_up_time= 'time',

out="No")

hl1 <- get_cox_weight(data=cleaned_v2_high_lipid1,

var='hei_2015_r ahei_2010_r dash_r Med_score_r',

covariate=cov_hlipid,

response='status',

follow_up_time= 'time',

out="No")

hl2 <- get_cox_weight(data=cleaned_v2_high_lipid2,

var='hei_2015_r ahei_2010_r dash_r Med_score_r',

covariate=cov_hlipid,

response='status',

follow_up_time= 'time',

out="No")

sub_status <- plyr::rbind.fill(data.frame(first_col="Age<65"),

age1,

data.frame(first_col="65=<Age"),

age2,

data.frame(first_col="正常/超重"),

bmi1,

data.frame(first_col="肥胖"),

bmi2,

data.frame(first_col="Male"),

gender1,

data.frame(first_col="Female"),

gender2,

data.frame(first_col="CVD:Yes"),

cvd1,

data.frame(first_col="CVD:No"),

cvd2,

data.frame(first_col="Diabetes:Yes"),

dabetes1,

data.frame(first_col="Diabetes:No"),

dabetes2,

data.frame(first_col="Dyslipidemia:Yes"),

hl1,

data.frame(first_col="Dyslipidemia:No"),

hl2) %>% dplyr::select(first_col,HRCI,P)

outrtf(sub_status,

cov="age race education marriage pir smoke energy egfr diabete cvd drink bmi",

path="")

####---------------------------------------CVD mortality----------------------------------------####

###screening

res1 <- get_cox_weight(data=cleaned_v2,

var='hei_2015_r ahei_2010_r dash_r Med_score_r',

covariate='',

response='status_cvd',

follow_up_time= 'time',

out="No")

res2 <- get_cox_weight(data=cleaned_v2,

var='hei_2015_r ahei_2010_r dash_r Med_score_r',

covariate='age race education marriage',

response='status_cvd',

follow_up_time= 'time',

out="No")

res3 <- get_cox_weight(data=cleaned_v2,

var='hei_2015_r ahei_2010_r dash_r Med_score_r',

covariate=cov_cvd,

response='status_cvd',

follow_up_time= 'time',

out="No")

res_sum_cvd <- cbind(res1[,c(1,2,4)],res2[,c(2,4)],res3[,c(2,4)])

##subgroup

cov_cvd_age <- "age race education marriage pir_r met smoke energy egfr drink diabete_status cvd_status cancer Anemia_trt gout copd Hemoglobin dialysis crp_r jiajian"

cov_cvd_bmi <- "age race education marriage pir_r met smoke energy egfr drink diabete_status cvd_status cancer Anemia_trt gout copd Hemoglobin dialysis crp_r jiajian"

cov_cvd_gender <- "age race education marriage pir_r met smoke energy egfr drink diabete_status cvd_status cancer Anemia_trt gout copd Hemoglobin dialysis crp_r jiajian"

cov_cvd_dibetes <- "age race education marriage pir_r met smoke egfr drink cvd_status cancer Anemia_trt gout copd Hemoglobin dialysis crp_r jiajian"

cov_cvd_hlipid <- "age race education marriage pir_r met smoke energy egfr drink diabete_status cvd_status cancer Anemia_trt gout copd Hemoglobin dialysis crp_r jiajian"

cov_cvd_cvd <- "age race education marriage pir_r met smoke energy egfr drink diabete_status cancer Anemia_trt gout copd Hemoglobin dialysis crp_r jiajian"

bmi1 <- get_cox_weight(data=cleaned_v2_BMI1,

var='hei_2015_r ahei_2010_r dash_r Med_score_r',

covariate=cov_cvd_bmi, response='status_cvd',

follow_up_time= 'time',

out="No")

bmi2 <- get_cox_weight(data=cleaned_v2_BMI2,

var='hei_2015_r ahei_2010_r dash_r Med_score_r',

covariate=cov_cvd_bmi,

response='status_cvd',

follow_up_time= 'time')

age1 <- get_cox_weight(data=cleaned_v2_age1,

var='hei_2015_r ahei_2010_r dash_r Med_score_r',

covariate=cov_cvd_age,

response='status_cvd',

follow_up_time= 'time')

age2 <- get_cox_weight(data=cleaned_v2_age2,

var='hei_2015_r ahei_2010_r dash_r Med_score_r',

covariate=cov_cvd_age,

response='status_cvd',

follow_up_time= 'time')

gender1 <- get_cox_weight(data=cleaned_v2_gender1,

var='hei_2015_r ahei_2010_r dash_r Med_score_r',

covariate=cov_cvd_gender,

response='status_cvd',

follow_up_time= 'time',

out="No")

gender2 <- get_cox_weight(data=cleaned_v2_gender2,

var='hei_2015_r ahei_2010_r dash_r Med_score_r',

covariate="age race education marriage pir_r met smoke egfr drink diabete_status cvd_status cancer Anemia_trt gout copd Hemoglobin dialysis crp_r jiajian",

response='status_cvd',

follow_up_time= 'time',

out="No")

dabetes1<- get_cox_weight(data=cleaned_v2_diabetes1,

var='hei_2015_r ahei_2010_r dash_r Med_score_r',

covariate=cov_cvd_dibetes,

response='status_cvd',

follow_up_time= 'time',

out="No")

dabetes2 <- get_cox_weight(data=cleaned_v2_diabetes2,

var='hei_2015_r ahei_2010_r dash_r Med_score_r',

covariate=cov_cvd_dibetes,

response='status_cvd',

follow_up_time= 'time',

out="No")

cvd1<- get_cox_weight(data=cleaned_v2_cvd1,

var='hei_2015_r ahei_2010_r dash_r Med_score_r',

covariate="age race education marriage pir_r met smoke energy egfr drink diabete_status cancer Anemia_trt gout copd Hemoglobin dialysis crp_r jiajian",

response='status_cvd',

follow_up_time= 'time',

out="No")

cvd2<- get_cox_weight(data=cleaned_v2_cvd2,

var='hei_2015_r ahei_2010_r dash_r Med_score_r',

covariate=cov_cvd_cvd,

response='status_cvd',

follow_up_time= 'time',

out="No")

hl1 <- get_cox_weight(data=cleaned_v2_high_lipid1,

var='hei_2015_r ahei_2010_r dash_r Med_score_r',

covariate=cov_cvd_hlipid,

response='status_cvd',

follow_up_time= 'time',

out="No")

hl2 <- get_cox_weight(data=cleaned_v2_high_lipid2,

var='hei_2015_r ahei_2010_r dash_r Med_score_r',

covariate=cov_cvd_hlipid,

response='status_cvd',

follow_up_time= 'time',

out="No")

sub_status_cvd <- plyr::rbind.fill(data.frame(first_col="Age<65"),

age1,

data.frame(first_col="65=<Age"),

age2,

data.frame(first_col="正常/超重"),

bmi1,

data.frame(first_col="肥胖"),

bmi2,

data.frame(first_col="Male"),

gender1,

data.frame(first_col="Female"),

gender2,

data.frame(first_col="CVD:Yes"),

cvd1,

data.frame(first_col="CVD:No"),

cvd2,

data.frame(first_col="Diabetes:Yes"),

dabetes1,

data.frame(first_col="Diabetes:No"),

dabetes2,

data.frame(first_col="Dyslipidemia:Yes"),

hl1,

data.frame(first_col="Dyslipidemia:No"),

hl2) %>% dplyr::select(first_col,HRCI,P)

outrtf(sub_status_cvd,

cov="age race education marriage pir smoke energy egfr diabete cvd drink bmi",

path="")

result <- rbind(res_sum,res_sum_cvd)

sub_result <- cbind(sub_status,sub_status_cvd)

openxlsx::write.xlsx(result,"logistic.xlsx")

openxlsx::write.xlsx(sub_result,"sub_result.xlsx")

screen <- cbind(screen1 %>% dplyr::select(first_col,HRCI,P),

screen2 %>% dplyr::select(HRCI11=HRCI,P11=P))

outrtf(screen,

cov="age race education marriage pir smoke energy egfr diabete cvd drink bmi",

path="")
